# Temperature Acclimation of the Picoalga *Ostreococcus tauri* Triggers Early Fatty-Acid Variations and Involves a Plastidial ω3-Desaturase

**DOI:** 10.3389/fpls.2021.639330

**Published:** 2021-03-19

**Authors:** Charlotte Degraeve-Guilbault, Nattiwong Pankasem, Maurean Gueirrero, Cécile Lemoigne, Frédéric Domergue, Tomonori Kotajima, Iwane Suzuki, Jérôme Joubès, Florence Corellou

**Affiliations:** ^1^Univ. Bordeaux, CNRS, Laboratoire de Biogenèse membranaire, UMR 5200, Villenave d’Ornon, France; ^2^School of Life and Environmental Sciences, University of Tsukuba, Tsukuba, Japan; ^3^Graduate School of Life and Environmental Sciences, University of Tsukuba, Tsukuba, Japan; ^4^Faculty of Life and Environmental Sciences, University of Tsukuba, Tsukuba, Japan

**Keywords:** temperature, microalgae (Mamiellophyceae), polyunsatutared-fatty-acid, octapentadecaenoic acid, desaturase, omega-3, transcription, *Ostreococcus tauri*

## Abstract

Alteration of fatty-acid unsaturation is a universal response to temperature changes. Marine microalgae display the largest diversity of polyunsaturated fatty-acid (PUFA) whose content notably varies according to temperature. The physiological relevance and the molecular mechanisms underlying these changes are however, still poorly understood. The ancestral green picoalga *Ostreococcus tauri* displays original lipidic features that combines PUFAs from two distinctive microalgal lineages (Chlorophyceae, Chromista kingdom). In this study, optimized conditions were implemented to unveil early fatty-acid and desaturase transcriptional variations upon chilling and warming. We further functionally characterized the *O. tauri* ω3-desaturase which is closely related to ω3-desaturases from Chromista species. Our results show that the overall omega-3 to omega-6 ratio is swiftly and reversibly regulated by temperature variations. The proportion of the peculiar 18:5 fatty-acid and temperature are highly and inversely correlated pinpointing the importance of 18:5 temperature-dependent variations across kingdoms. Chilling rapidly and sustainably up-regulated most desaturase genes. Desaturases involved in the regulation of the C18-PUFA pool as well as the Δ5-desaturase appear to be major transcriptional targets. The only ω3-desaturase candidate, related to ω3-desaturases from Chromista species, is localized at chloroplasts in *Nicotiana benthamiana* and efficiently performs ω3-desaturation of C18-PUFAs in *Synechocystis* sp. PCC6803. Overexpression in the native host further unveils a broad impact on plastidial and non-plastidial glycerolipids illustrated by the alteration of omega-3/omega-6 ratio in C16-PUFA and VLC-PUFA pools. Global glycerolipid features of the overexpressor recall those of chilling acclimated cells.

## Materials and Methods

All chemicals were purchased from Sigma Chemical (St. Louis, MO, United States), when not stated otherwise.

### Biological Material and Cultures

*Ostreococcus tauri* (clonal isolate from OtH95) wild-type and transgenics were grown and monitored by flow cytometry as previously described (Degraeve-Guilbault et al., 2017). Vancomycin (1 mg/ml) was used to reduce bacterial contamination to less than 1% before experiments. Artificial sea-water base contained either 5 μM NaH_2_PO_4_ (phosphate limitation) or 35 μM. Cultures were grown in incubator-shaker (New Brunswick Innova 42R) with constant agitation (80 RPM) under white light (75 mmol photons m-2 s-1, 6 × T8 fluorescent bulbs 15 Watt each (Sylvania Gro-Lux). For screening of FA of *O. tauri* transgenics, cells were grown in T25 aerated culture flasks (Sartstedt, Nümbrecht, Germany) at 20°C. For *O. tauri* lipid analysis cells were grown in 200 mL of medium in 500 mL Erlenmeyer flasks. For temperature shift experiments cells were grown in either Erlenmeyer flasks or aerated T75 vertical flasks (100 mL medium in 250 mL flasks). *O. tauri* were acclimated at least for 10 generations (sub-cultivated twice) at a given temperature. *Synechocystis* sp. PCC6803 was grown accordingly to Kotajima et al. (2014). *Nicotiana benthamiana* plants were cultivated in a greenhouse under controlled conditions (16–8 h photoperiod, 25°C). *Agrobacterium tumefaciens* strain GV3101 was grown in Luria Broth medium at 30°C as previously described (Degraeve-Guilbault et al., 2020).

### Sequences Analyses

Sequences of putative ω3-desaturases were retrieved from genomic and transcriptomic data from NCBI and those from Mamiellophyceae species were manually checked for completion of Nt sequences; cTP were predicted from PredAlgo; alignment was performed using Snapgene trial version (Clustal omega). A codon-optimized sequences without the cTP (sequence start MTYNET) was used for expression *Synechocystis* sp. PCC6803 (Genewiz, Europe).

### Cloning Strategy

The *O. tauri* ω3-desaturase full-length sequence was amplified by PCR cycles from cDNA matrix using Q5^®^ Polymerase by two-step PCR (5′-ATGCGCGCCGCGACGTC-3′ and 5′-CTAGTCGCCCCGCTCCCAGAC-3′), cloned in pGEM^®^-T Easy (Promega, Madison, WI, United States) and sequenced (Genwiz, Leipzig, Germany). Amplification from plasmid DNA was achieved with adapted primers to allow further cloning in pOtox-Luc (Moulager et al., 2010) (Restriction sites *Apa*I, *Avr*II) and using Gateway^®^ system according to manufacturer instruction (pDONR 221, pVT102-U-GW for *Saccharomyces cerevisiae* and pK7W2G2D or pK7YWG2 for *N. benthamiana*). Primers are provided in Supplementary Tables.

Overexpression in *Synechocystis* sp. PCC6803 was performed using the pTHT2031S vector after ligation to introduce the synthetic gene using the In-Fusion^®^ HD cloning kit (Takara Bio, Kusatsu, Japan). Primers are available from the Supplementary Data File.

### RNA and cDNA Preparation and Quantitative RT-PCR Analysis

For every RNA extraction FAs were analyzed in parallel. RNeasy-Plus Mini kit (Qiagen, Hilden, Germany) was used for RNA purification; DNase I was used to remove contaminating DNA (DNA-free kit, Invitrogen, Carlsbad, CA, United States) and cDNA obtained using the reverse transcription iScript^TM^ supermix kit (Bio-Rad, Hercules, CA, United States). Real-time RT quantitative PCR reactions were performed in a CFX96^TM^ Real-Time System (Bio-Rad) using the GoTaq^®^ qPCR Master mix (Promega, Madison, WI, United States). Bio-Rad CFX Manager software was used for data acquisition and analysis (version 3.1, Bio-Rad). Ct method was used to normalized transcript abundance with the references mRNA *EF1*α (elongation factor), *CAL* (calmodulin), and *ACTprot2* (Actin protein-related 2). PCR efficiency ranged from 95 to 105%. Primers are available from the Supplementary Data File.

### Genetic Transformation

*Ostreococcus tauri* transformation was achieved using the pOtOXLuc vector and electroporation and transgenics were pre-screened accordingly to their luminescent level as previously described (Degraeve-Guilbault et al., 2020). Control lines are transgenics of empty vectors.

*Nicotiana benthamiana* were transformed by agroinfiltration of leaves from five-week old plants as previously described (Degraeve-Guilbault et al., 2020). Co-infiltration of RNA-silencing inhibitor P19 (equal volume of a bacterial suspension harboring pBin61-P19), was used in all experiments (Shah et al., 2013). DNA constructs were transferred by electroporation into the *Agrobacterium tumefaciens* GV3101 strain. Briefly, *A. tumefaciens* transformants were selected with antibiotics (gentamycin 25 μg/mL with spectinomycin 100 μg/mL or kanamycin 50 μg/mL). *A. tumefaciens* transformants were grown overnight, diluted to an optical density at 660 nm of 0.1, and grown up to 0.6–0.8. Cells were re-suspended in 5 mL sterilized H_2_O for a final OD of 0.4 and 0.2 for overexpression and subcellular localization experiments, respectively. and 1 mL was agroinfiltrated using a synringe without needle. Plants were analyzed 2 and 5 days after *A. tumefaciens* infiltration for subcellular localization experiments and for overexpression, respectively.

*Synechocystis* sp. PCC6803 transformation was achieved by homologous recombination. Briefly, the plasmid was transformed into ten-time concentrated cells collected at mid-log phase. Subsequently, the cell was incubated at 30°C under white fluorescent lamps for 16–18 h and selected by 25 μg/mL chloramphenicol and 5 μg/mL spectinomycin on BG-11 solid media (1.5% w/v Bacto-agar).

### Lipid Analysis

For all organisms, fatty acid analyses and for *O. tauri* further lipid analysis were achieved accordingly to Degraeve-Guilbault et al. (2017). Organic solvents all contained butylhydroytoluene as an antioxidant (0.001%) and glassware was used. To gain resolution on *O. tauri* FA analysis, a minimum of 50 mL culture (approx. 1.5 × 10^9^ cells) was pelleted and extracted (glass beads beating and 1 h at 80°C) in 1 mL acidic methanol (2% v/v H_2_SO_4_) containing heptadecanoic acid (2 or 10 μg/ml) as internal standard; phase separation was achieved using 1 mL of NaCl 2.5% (or water for *O. tauri*) and 1 mL of hexane. The upper phase was collected in a new tube and concentrated to 100 μL under nitrogen stream. Four μL were injected for GG-FID analysis (Hewlett-Packard 5,890 series II, Agilent, Waldbronn, Germany) on a15 m × 0.53 mm × 1.2 μ Carbowax column (Altech, Deerfield, IL, United States). This procedure allowed for increasing the resolution of minor FA detection without any column saturation with major FA. Lipid extraction was performed as previously described (Degraeve-Guilbault et al., 2020). Briefly, the material was extracted using glass beads in chloroform:methanol (2:1 v/v), pelleted and extracted again until no pigment could be extracted. Phase separation was performed adding 0.5 v of NaCl 0.9%. Lipid developments were achieved by HP-TCL under 33% humidity in the ADC2-chamber system, (CAMAG). For *O. tauri* polar lipid were separated using methyl acetate/isopropanol/chloroform/methanol/KCl 0.25% (25:25:25:10:4 v/v/v/v) and neutral lipid using hexane/diethyl-ether/glacial-acetic-acid (60:10:1.22 v/v/v). For *Synechocystis* sp. PCC6803, polar lipids were separated using chloroform/methanol/glacial acetic acid/water (85:12:12:1 v/v/v/v). Lipids were stained with a solution of 0.02% primuline in 80:20 acetone/water (deeping for 1 min, air dried for 20 min).

### Confocal Microscopy

Live cell imaging was performed using a Leica SP5 confocal laser scanning microscopy system (Leica, Wetzlar, Germany) equipped with Argon, DPSS, He-Ne lasers, hybrid detectors, and 63x oil-immersion objective. *N. benthamiana* leave samples were transferred between a glass slide and coverslip in a drop of water. Fluorescence was collected using excitation/emission wavelengths of 488/490–540 nm for chlorophyll, 488/575- 610 nm for YFP, and 561/710- 740 nm for m-cherry. Co-localization images were taken using sequential scanning between frames. Experiments were performed using strictly identical confocal acquisition parameters (e.g., laser power, gain, zoom factor, resolution, and emission wavelengths reception), with detector settings optimized for low background and no pixel saturation.

### Statistical Analyses

GraphPad Prism version 9.0.0 for Windows (GraphPad Software, San Diego, CA, United States^1^), was used to compute statistical analysis following the recommendation of the user guide. PCA analyses was performed using the standardize method with parallel analysis option. The unpaired *t*-test with Welch correction (no assumption made about the variance of each group) was used when normality could be assessed otherwise the non-parametric Mann–Whitney test was used. Note that only the Shapiro–Wilk test for normality provided results when *n* = 3.

## Introduction

Microalgae are key primary producers of polyunsaturated fatty-acids (PUFAs) including very-long chain PUFAs (VLC-PUFAs) commonly found in marine species (Khozin-Goldberg et al., 2016; Jonasdottir, 2019). Omega-3 PUFAs (ω3) largely predominate over omega-6 PUFA (ω6). Transfer and accumulation of PUFAs through the food-web is essential to support fundamental processes including fertility, development immunity at many trophic levels (Kainz et al., 2004). The synthesis of PUFAs in glycerolipids highly relies on desaturases (Des) that sequentially add *cis-*double bond at specific locations (stereospecificity and regiospecificity) (Shanklin et al., 2009). Des are called after their regiospecificity; for instance, Δ6-Des introduce a double bond in the acyl chain at the sixth carbon from the carboxyl-end (Δ-end) while ω-3-Des convert ω3 to ω6 (methyl end). Desaturases specificity also relies on the acyl-carrier, which can be soluble (Co-enzymeA and acyl-carrier-protein, ACP) or membranous (lipid). Exception made of the stearoyl-CoA Des, primitive eukaryotes (plants, worms…) displays acyl-lipid desaturases. In contrast to “higher” eukaryotes, they retained the ability to convert ω6 to ω3. The interplay of desaturases activity within and between the ω3 and ω6 pathways is assumed to be key for PUFA composition of microalgae and consequently for the well balance of ω6/ω3 in upper trophic levels (Galloway and Winder, 2015; Sun et al., 2019).

Phylogenetic markers could be identified despite the important variation of FA composition, including across species from a same taxa (Lang et al., 2011; Jonasdottir, 2019). The ω3 16:4^Δ4,7,10,13^ (16:4n3) is the signature of Chlorophyta (Archaeplastida kingdom, “green lineage”) while VLC-PUFAs such as 20:5^Δ5,8,11,14,17^ (20:5n3) and 22:6 ^Δ4,7,10,13,16,19^ (22:6n3) are hallmarks of the Chromista kingdom. The peculiar 18:5^Δ3,6,9,12,15^ (18:5n3), initially characterized from dinoflagellates (Dinophyta), is widespread in Chromista species including haptophytes and some raphidophytes (Ochrophyta) (Jonasdottir, 2019). 18:5n3 is only found in some classes of Chlorophyta that emerged early in the green lineage such as Pyramimonadphyceae and Mamiellophyceae. This highly unsaturated C18 has been reported to be rapidly metabolized in fish and give rise to 20:5n3 (Ghioni et al., 2001).

Abiotic stresses have major impact on microalgae FA composition (Los et al., 2013; Khozin-Goldberg et al., 2016; Kugler et al., 2019). In the context of climate change, it is all the more important to understand how temperature influences the fatty acid composition of key marine phytoplankton species. Temperature is recognized as a major cue for the regulation of PUFA composition of all organisms including cyanobacteria and microalgae (Nishida and Murata, 1996; Boelen et al., 2013; Kotajima et al., 2014; Aussant et al., 2018). Increasing unsaturation degree are usually inversely correlated with temperature. However, for microalgae the impact of temperature on FA-profile appears highly dependent on the species. Important studies using *Bacillus subtilis* and *S. cerevisiae*, demonstrated that changes in the membrane state regulate FA desaturase expression allowing for the acclimation of membrane fluidity to occur (homeoviscous response) (Mansilla et al., 2008; Ernst et al., 2016). However, only desaturases introducing monounsaturation and monounsaturated FAs have been clearly demonstrated to be involved in both responses to membrane state and membrane fluidity changes. In contrast, the role of PUFAs for membrane organization appears much subtle. In plants, temperature regulation of desaturase has also been shown to occur at post-transcriptional levels (Gibson et al., 1994; Matsuda et al., 2005; Los et al., 2013).

*Ostreococcus tauri* is a representative species of the class Mamiellophyceae (Chlorophyta) (Chrétiennot-Dinet et al., 1995). Mamiellophyceae usually predominate marine picophytoeukaryote communities which have a fundamental role in coastal ecosystems (Massana, 2011; Rii et al., 2016). *O. tauri* and related species display a unique FA composition gathering features from the Archaeplastida and Chromista kingdoms. Together with the saturated FAs (SFA) 14:0 and 16:0, the ω3 16:4, 18:3, 18:4, 18:5, and 22:6 are the main FAs (Degraeve-Guilbault et al., 2017). The distribution of these PUFAs is strikingly clear-cut: C18-PUFAs prevail in plastidic lipids, 18:5n3 being restricted to galactolipids, and VLC-PUFAs are exclusively found in the extraplastidic lipids. Non-plastidic lipids are the betain lipid diacylglyceryl-hydroxymethyl-trimethyl-β-alanine (DGTA) and phosphatidyldimethylpropanethiol (PDPT), which are usually reported in Chromista species. We previously showed that nutrient availability importantly impacted the ratio of Δ6-Des substrates/products in plastidial C18-PUFAs and identified the two first plastidic Δ6-Des, one of which was transcriptionally repressed under phosphate starvation (Degraeve-Guilbault et al., 2017, 2020).

In the present work we studied the impact of temperature changes on *O. tauri* FA profile and glycerolipids content. Early changes in Des expression upon thermal shift were investigated. The ω3-Des from *O. tauri* was functionally characterized by overexpression in *N. benthamiana*, *Synechocystis* sp. PCC6803 (*Synechocystis* thereafter) and *O. tauri* giving insight into its involvement in temperature responses.

## Results

### Temperature Impact on Glycerolipid FA Composition

**FIGURE 1:**
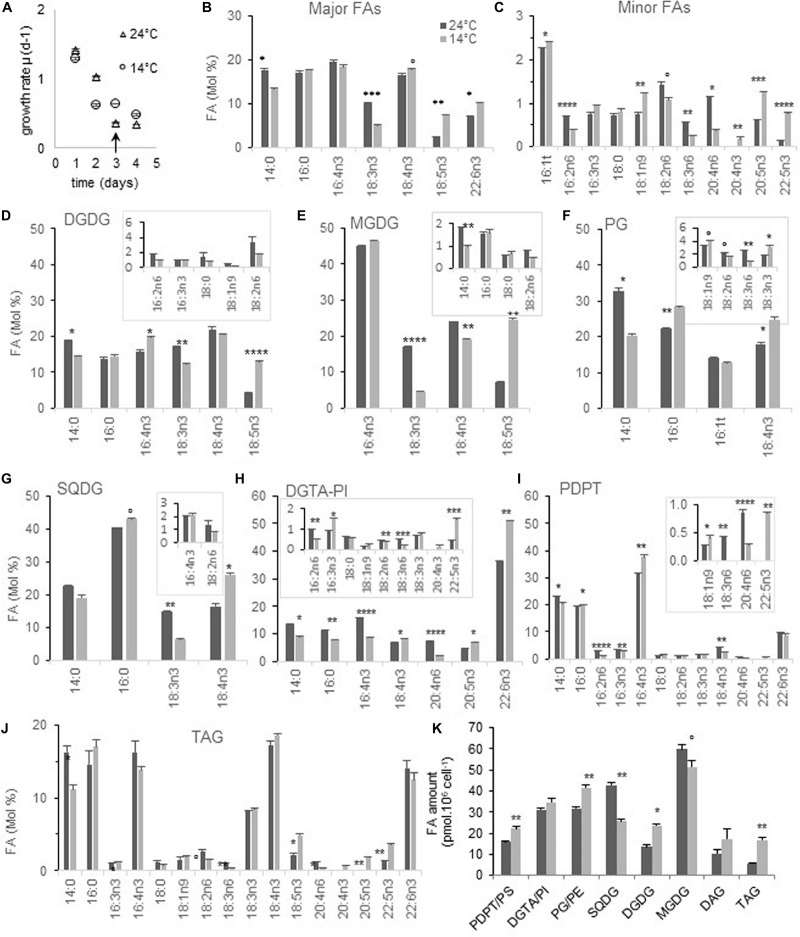
FA and glycerolipid profiles from cells acclimated to 24 and 14°C. Cells were grown under continuous light and acclimated at each temperature before sampling. FAs ≤ 0.5% at both temperatures are not plotted except for 20:4n3 and PDPT. **(A,B)** Global FA profiles from glycerolipid analysis. **(C)** Growth rate of corresponding cultures; arrows indicate the sampling time. **(D–K)** Glycerolipid FA composition. Minor FAs are highlighted in framed inserts. Means and standard errors to the mean (SEM) from biological triplicate are shown. Marks correspond to statistical significant differences by unpaired *t*-test (°*P* < 0.02, *P < 0.01, **P < 0.001, ***P < 0.0001, and ****P ≤ 0.00001).

We previously showed that during *O. tauri* batch-growth the proportion of 18:3n3 was readily increased at the expense of 18:4n3 and therefore might mask some specific impact of temperature on these FAs (Degraeve-Guilbault et al., 2017). Consequently, preliminary experiments were conducted to determine high and low temperatures for which minimal differences were observed with regards to the growth rate between temperatures and maximal differences for FA. Temperatures tested corresponded to those were *O. tauri* has been detected in the environment (Supplementary Figure 1; Limardo et al., 2017). In our conditions, cell-growth and/or viability appeared to be impaired at 10 and 32°C. Growth did not appear to be impacted at 15 and 25°C and monitoring growth in more details at 14°C and 24°C confirmed that acclimated cells displayed a similar daily growth rate with a μmax (14°C 0.91 ± 0.1 d^–1^ and 24°C 0.99 ± 0.146 d^–1^) that was comparable to the one previously observed at 20°C (0.99 ± 0.006) (Degraeve-Guilbault et al., 2017; Supplementary Figure 1A and Figure 1A). Principal component analysis of FA and temperature values from preliminary experiment unveiled an inversed correlation of 18:5n3 and temperature, and a direct correlation of most ω6 FAs and 18:3n3 (Supplementary Figures 1C,D). To gain further insight into FA variations, the glycerolipid FA profile of cells acclimated at 14 and 24°C collected in mid-exponential growth (3.5 × 10^7^ cell mL^–1^) was analyzed (Figure 1). The proportion of most ω3-PUFAs was overall increased at the expense of ω6-PUFAs at 14°C. The proportion of 14:0 was also reduced. It was particularly noteworthy that 18:3n3 and 18:5n3 varied in a reverse way while 18:4n3 remained overall stable (Figure 1B). the most statistically relevant differences were detected for the ω3 18:3, 18:5, 20:4, 20:5, 22:5, and for the ω6 16:2, 18:3 as well as for the monounsaturated FA 18:1n9; an obvious drop occurred for the ω6 18:2 and 20:4 though computed *P* values were higher (Figure 1C).

Glycerolipid analysis from acclimated cells showed that the proportion of 18:5n3 was increased at low temperature in both MGDG and DGDG (Figures 1D,E). For MGDG this seemed to occur at the expense of both 18:3n3 and 18:4n3 while only at the expense of 18:3n3 in DGDG. The 18:4n3 proportion was higher in both SQDG and PG which are lacking 18:5n3, and this increase appeared to occur at the expense of 18:3n3 in SQDG (Figures 1F,G). A higher proportion of 22:6n3 was observed in DGTA but neither in PDPT/PS nor in TAG (Figures 1I,H,J). On the other hand, variations of other minor VLC-PUFAs such as 20:4n6, 20:5n3, and 22:5n3 were reverberated into TAG, in which 20:4n3 was also detected only at low temperature (Figure 1J). These changes translated into a higher unsaturation degree of the bulk of glycerolipids, including TAG, as well as a higher ω3/ω6 ratio in most glycerolipids, that was the highest for PG (Supplementary Figures 2A,B).

The glycerolipid composition was also impacted (Figure 1K and Supplementary Figure 1C). Considering the cellular amount, DGDG was increased at the expense of MGDG at 14°C (Figure 1K). This feature has been reported decades ago in both higher plants and algae grown at low temperature and is assumed to be related to the stabilization of plastid membranes in response to various stresses including phosphate deprivation (Lynch and Thompson, 1982; Kuiper, 1985; Li and Yu, 2018). More surprisingly, the amount of SQDG was significantly reduced while that of phospholipids increased, a feature recalling increased phosphate availability, though not coherent with the variation observed in galactolipids (Van Mooy et al., 2009). Noteworthy TAG were twice as abundant at low temperature.

Altogether, our results indicate that low temperature acclimation correlates with an overall increase of ω3-PUFAs and a concomitant decrease of ω6-PUFAs in all structural glycerolipids. A drop of 18:3n3 occurred in all plastidial lipids and was concomitant of a rise of 18:5n3 in galactolipids whereas 18:4n3 was increased only in PG and SQDG.

### Kinetics of FA Desaturation Upon Temperature-Shift

From experience we know that *O. tauri* rhythms are readily synchronized by external cues (Moulager et al., 2007; Monnier et al., 2010). In order to gain the most accurate insight into the kinetics of changes upon temperature shift, the cultures were synchronized by light-dark cycles (L/D) of 18–6 h. This experimental design aimed to restrain time resetting and cell re-synchronization by temperature shift. Indeed, continuous light does not abolish circadian rhythms but merely result in progressive desynchronization of rhythms between individual cell (Bieniawska et al., 2008). As temperature is a strong cue for time resetting, the cells switched to a novel temperature are most likely resynchronized while the control cell are not (McClung and Davis, 2010). This results in comparing different internal times. In contrast, under diurnal cycles internal rhythms and entrained, i.e., synchronized and set on the external time; temperature changes do not change the phase of the rhythms. Because temperature responses were reported to be augmented in the morning and since in *O. tauri* desaturase expression is known to peak from late night to mid-day, temperature-shifts were achieved [4.5 h after light on (T0)] (Supplementary Figure 3).

**FIGURE 2:**
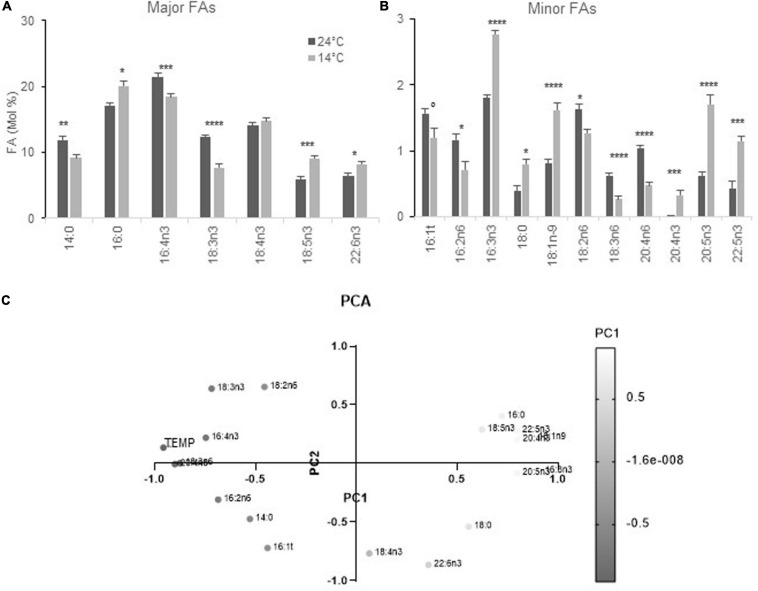
FA profiles from cells acclimated to 24 and 14°C under diurnal conditions. Cells were grown under light-dark cycles (16–8 h). **(A,B)** FA-profile from cells collected at 4–4.5 h after light on. Means and standard errors to the means of 15 samples (extracted from four independent experiments) are shown. Marks correspond to statistical significant differences by unpaired *t*-test (°*P* < 0.05, *P < 0.01, **P < 0.001, ***P < 0.0001, and ****P < 0.00001). **(C)** Principal component analysis (PCA) of FAs and temperatures from the same data set. PC1 corresponds to 50.27% of the variance and PC2 to 19.96% (cumulative proportion of the variance 70.23%). The gray gradient applies for the value on PC1 the lightest being the lowest.

Cells acclimated at 14 and 24°C and collected at the time of the temperature shift displayed FA differences that were closely related to those observed under continuous light, though the *P* varied, with the further variation of 16:4n3 that was lower at 14°C (Figures 2A,B). Principal component analysis was used to test the relationship between FA variations and temperature in this homogeneous data set; it unveiled that minor ω3 PUFAs as well as 18:5n3, 16:0, 18:1, and were related and negatively correlated to temperature. In contrast, 20:4n6, 18:3n6 as well as 16:4n3 displayed correlation scores that were close to the temperature variable while 18:2, 16:2 as well as 18:3n3 variations were also explained by the second principal component (Figure 2C).

**FIGURE 3:**
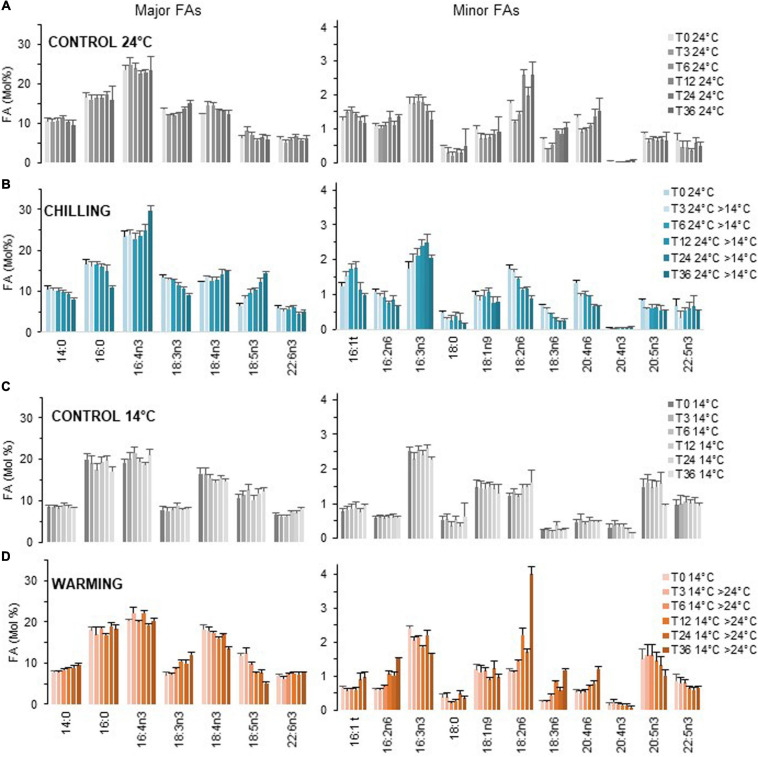
Kinetics of FA profile variations upon chilling and warming. Major and minor FAs are represented on separate panels. Cultures grown under light/dark cycles (16/8 h) at 24°C **(A)** and 14°C **(C)** were transferred at 4.5 h after light on (T0) to 14°C [chilling, **(B)**] and 24°C [warming, **(D)**] respectively. Means and standard errors of biological replicate from at least two independent experiments (T36 chilling triplicate from one experiment).

The swiftness of FA-variations upon chilling (24–14°C) and warming (14–24°C) was investigated achieving early sampling times after the temperature and following variations up to 36 h (Figure 3). The data were averaged from independent experiments. As the proportion of 18:3n3 and 18:4n3 were slightly different between experiments (due to the nutrient state), an individual kinetics is further provided as Supplementary Data, in order to better highlight the most earlier changes (Supplementary Figure 4).

#### Fatty-Acid Variations

##### Acclimated Cells

Over the 36 h sampling period the proportion of major FAs remained in average stable while that of the ω6 18:2, 18:3, and 20:4 progressively increased at 24°C (Figures 3A,C). The proportion 18:2n6 was further increased at dark transitions (T12, T36) (see also Supplementary Figure 4).

##### Temperature Shifts

Variations upon chilling were detected as early as 3 h, in particular for the minor FAs 18:2n6 and 18:3n6 and the major FA 18:5n3 (Figure 3B). It was striking that under chilling, the proportion of 18:3n3 was gradually decreased over time which contrasted to all other conditions. The proportion of 18:4n3 remained rather stable. Chilling also resulted in the gradual increase of 16:3n3 and was concomitant of a decrease of 16:2n6. For VLC-PUFAs, the proportion of 20:4n6 was progressively lowered while the increase of either 20:5n3 and/or 22:5n3 were not obvious from the pool of all data but detected in individual experiments (Supplementary Figure 4). Noteworthy, 22:6n3 was poorly/not increased over the sampling period and the minor FA 20:4n3 was detected only in cells acclimated to low temperature (Figure 3 and Supplementary Figure 4). Conversely, warming triggered changes that were unambiguously detected after 6 h and were reversed compared to chilling; those included the increase of 18:3n3 that paralleled the decrease of both 18:4n3 and 18:5n3 as well as the increase of the ω6 (16:2,18:2, 18:3, and 20:4) that were concomitant with the decrease of minor ω3 (Figure 3D). Note that the 20:4n3 was still detected 36 h after transfer to high temperature.

In summary, chilling and warming triggered FA changes detected as early as 3–6 h after the shift, respectively. The patterns were reversed between chilling and warming and impacted the overall ω3/ω6 ratio. The earliest and most obvious variations were observed for ω6-C18-PUFAs and 18:5n3. Progressive variations further occurred in minor FAs including 16:2n6, 16:3n3, 20:4n6, and 20:5n3 and/or 22:5n3. The variations of 18:3n3 mirror the changes observed in 18:5n3 whereas the proportion 18:4n3 was rather stable. Finally, accumulation of 22:6n3 and 20:4n3 occurred at long term. These observations strongly suggested the involvement of ω3-desaturation in early temperature responses.

#### Desaturase Transcript Variations

**FIGURE 4:**
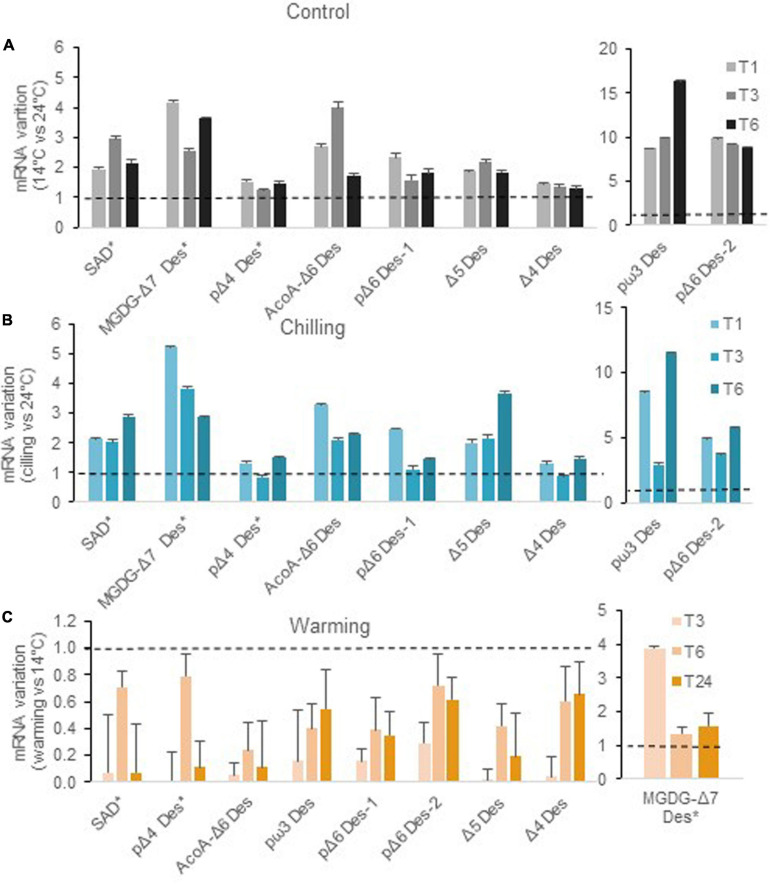
Variations of desaturase transcript abundance upon chilling and warming relative to control cultures. Culture conditions were the same as in Figure 2. Plotted values correspond to the ratio of transcript abundance at 14°C relative to 24°C for controls **(A)**, and ratio of transcript abundance in cells shifted to either low **(B)** or high **(C)** temperature relative to cells at the initial temperature. Errors bars are standard deviations calculated following the error propagation rules using the formula SD_*x/y*_ = √(SD_*x*_/x)^2^ + (SD_*y*_/y)^2^. Relative transcript abundance is available from Supplementary Figure 5.

In order to gain insight into early transcriptional control possibly involved in the dynamics of FA-changes, the expression of desaturases most relevant to the changes observed at the FA level were monitored (Figure 4 and Supplementary Figure 5). Since FA variations upon chilling were detected as early as 3 h, desaturase expression upon chilling was monitored as earlier as 1 h after chilling.

Except for the ER and plastidial Δ4-Des, all desaturase transcripts were higher in cells acclimated or transferred to 14°C (Figures 4A,B and Supplementary Figure 5A). Values were unambiguously increased as early as 1 h after the shift. The highest differences were observed for the putative ω*3-Des* and the *plastidial*Δ*6-Des-2* (previously referred to as Ot10) whereas *p*Δ*6-Des-1* (previously Ot05) was moderately upregulated though its expression was definitely higher in cells acclimated to low temperature. Let us recall here, that pΔ6-Des2 has been recently shown to preferentially impact ω6-C18PUFAs, PG as well as highly unsaturated ω3-galactolipid species while the overexpression of *p*Δ*6-Des-1* had a much broader impact and was highly active on ω3-substrates (Degraeve-Guilbault et al., 2020). Early up-regulation was also unambiguous for the *Acyl-CoA*Δ*6-Des* and Δ*5-Des*, coding both for ER Des, as well as for the *MGDG*Δ*7-Des* and the *stearoyl-ACP desaturase* putative ortholog (*SAD*). Note that, similar trends were monitored under continuous light though both *Acyl-CoA*Δ*6-Des* and *p*Δ*6-Des1* underwent higher activation; this suggested that, under L/D, the full activation of these genes in the morning could be limited by a circadian control (circadian gating) (Supplementary Figure 6). Conversely, warming resulted in an overall reduction of desaturase transcript levels especially at 3 h after the temperature-shift, with the notable exception of the putative *MGDG*Δ*7-Des* whose expression appeared transiently increased (Figure 4C). Over-time, desaturase gene downregulation was more conspicuous for the *Acyl-CoA*Δ*6-Des*, as well as for Δ*5-Des, p*Δ*6-Des-1*, and the putative *p*ω*3-Des* whose expressions were reduced by more than half.

Overall, our results indicate a tight and inverse control of the expression of most desaturase genes by temperature. The *Acyl-CoA* Δ*6-Des*, the putative ω*3-Des* and the Δ*5-Des* expressions appeared to be importantly regulated in both cases. Interestingly for the plastidial Δ6-desaturases, the expression of the *p*Δ*6-Des2* was importantly increased upon chilling whereas for warming, *p*Δ*6-Des1* transcript level was reduced to a greater extent compared to *p*Δ*6-Des2*.

### Functional Characterization of the Putative ω-3-Des

As both FA and transcriptional variations strongly suggested the involvement of the ω3-Des in the temperature acclimation process, we next focused on the only ω3-Des candidate found in *O. tauri* (Otpω3-Des) and related species (Degraeve-Guilbault et al., 2020). These putative ω3-Des have been reported to be orthologous of the Δ15-Des of *Emiliania huxleyi* (EhΔ15-Des) and cluster apart from previously characterized Δ15-Des. The authors hypothesized that EhΔ15-Des was involved in the accumulation of 18:5n3 that occurs in *E. huxleyii* at low temperature (Kotajima et al., 2014).

#### Sequence Features

We used sequences of functionally characterized ω3-Des from the green lineage and sequences with homology to the Otpω3-Des in different lineages to construct a phylogenetic tree (Supplementary Figure 7A). Our analysis confirmed that ω3-Des sequences from Mamiellophyceae were unrelated to green ω3-Des and clustered with desaturase sequences from the Chromista kingdom including the ω-3-Des from *Thraustochytrium* sp. shown to accept C20-FAs as substrates (Meesapyodsuk and Qiu, 2016). Sequence alignment of putative ω-3 Des from Mamiellophyceae species highlighted three typical His-boxes encompassing the motifs HHTCH, HNHLHH, and YQIEHH conserved in orthologs from haptophytes, diatoms, dinoflagellates, and labyrinthulomycetes and divergent form the motifs HXXXH, HXXHH, and HXXHH considered as hallmarks of eukaryotic ω-3 Des (Supplementary Figure 7B; Wang et al., 2013; Kabeya et al., 2018). In addition, all ω3-Des sequences from Mamiellophyceae encompassed a predicted chloroplastidic targeting peptide (cTP) upstream of highly conserved sequence starting with a methionine (Supplementary Figure 8). According to the PredAlgo prediction software the cTP score of Otpω3-Des was the highest among all desaturases (4.53/5) (Tardif et al., 2012; Degraeve-Guilbault et al., 2020).

#### Overexpression of the Putative ω3-Des in Heterologous Hosts

**FIGURE 5:**
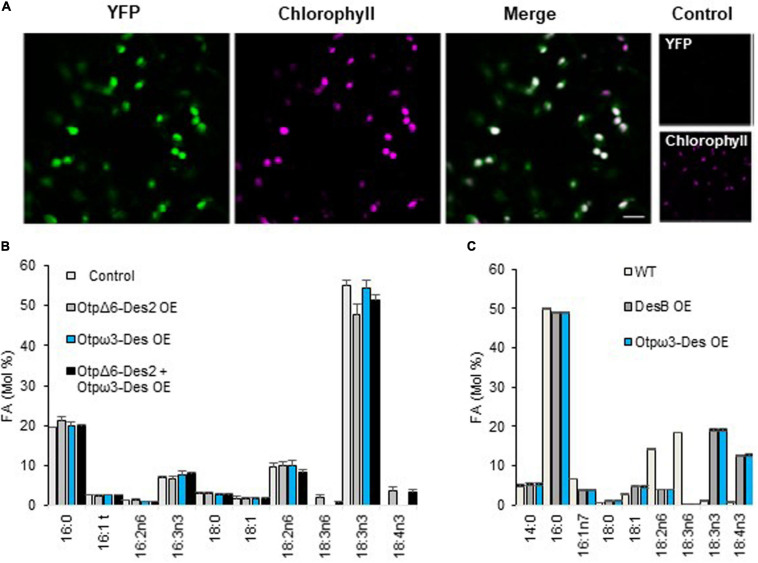
Localization and activity of the *O. tauri* putative ω3-Des in heterologous hosts. **(A)** Confocal Microscopy of *N. benthamiana* transient overexpressing the putative ω3-Des C-terminal fused to YFP (YFP) (100% for 23 cells). Fluorescence from YFP, chlorophyll and cross bleeding with identical parameter (control). **(B)** Overexpression of the label-free putative ω3-Des (pω3-Des) in *N. benthamiana.* The overexpression of the plastidial *O. tauri pΔ6-Des2* (pΔ6-Des2) is used to highlight the ω3-activity by competing for their common substrate 18:2n6. Means and standard errors of n replicate from two independent experiments are shown; P19 transgenics (control *n* = 5), pΔ6-Des2 OE (*n* = 7), pω3-Des OE (*n* = 6), pΔ6-Des2 + pω3-Des OE (*n* = 17). **(C)** Overexpression of the putative ω3-Des truncated for the cTP in *Synechocystis* PCC6803. A line overexpressing the native ω3-Des (DesB OE) was used as positive control. Cells were grown at 32°C, temperature at which the expression of *desB* is repressed in the wild-type. 14:0, 16:1n7, and 18:1 that represent each about 5% and did not varied are not represented. Means and standard errors of three independent experiments are shown.

Transient expression of the *Otp*ω*3* fused to YFP in *N. Benthamiana* resulted in a clear labeling of chloroplasts (Figure 5A). Overexpression of the label free *Otp*ω*3-Des* had no detectable impact on the overall FA-profile (Figure 5B). We reasoned that the endogenous ω3-Des activity (FAD3/FAD7/FAD8) may mask the activity of the *Otp*ω*3-Des*. To possibly unmask this activity, the *O. tauri p*Δ*6-Des2 (Otp*Δ*6-Des2)* was used. We previously showed that pΔ6-Des2 displays a preference for ω6-C18-PUFA; it was assumed to compete for the 18:2n6 the natural substrate of plant ω3-Des, thereby reducing the amount of 18:3n3 but allowing 18:4n3 production through 18:3n6 ω-3 desaturation (Degraeve-Guilbault et al., 2020). As expected, *Otp*Δ*6-Des2* overexpression in *N. benthamiana* resulted in the production of 18:3n6 and 18:4n3 while 18:3n3 was in average concomitantly decreased compared to the control lines, though the difference was estimated to be poorly relevant (*t*-test, *P* = 0,021*)*; co-expression of *Otp*Δ*6-Des2* OE and *Ot*pω3-Des lowered the proportion of 18:3n6 (*t*-test *P* = 0,035) and that of 18:3n3 was in average increased though it did not appear to be statistically relevant. These subtle FA-variations were coherent with *Ot*pω3-Des triggering the desaturation of 18:3n6 and/or competing with *Otp*Δ*6-Des2* for 18:2n6, thereby indirectly reducing the proportion of 18:3n6. The absence of clear variations of ω3-end-products 18:3n3 and 18:4n3 might result from compensatory regulations of the endogenous ω3-Des activities. In order to circumvent interference of endogenous ω3-Des activities, *Synechocystsis* sp. PCC6803 (*Synechocystis* thereafter) was chosen to overexpress *Otp*ω*3-Des*. *Synechocystsis* displays one ω3-Des (DesB) whose expression is strongly inhibited at 32°C, precluding the production of 18:3n3 and 18:4n3 from 18:2n6-PG/SQDG species and 18:3n6-galactolipid-species, respectively (Figure 5C; Sakamoto et al., 1994). Heterologous gene expression in *Synechocystis* has the further advantage of being driven from the same insertion site (homologous recombination) (Williams, 1988). At 32°C, the *desB* and *Otp*ω*3-Des* overexpressors (OE) produced 18:3n3 and 18:4n3 in a similar way. Both OE accumulated 18:4n3 in galactolipids and 18:3n3 in PG and SQDG indicating that *Otp*ω*3-Des* accepted equally well galactolipids, PG and SQDG as substrates (Supplementary Figure 9). Altogether these results demonstrated that *Ot*pω3-Des is a plastid located desaturase which efficiently converts ω6-C18-PUFAs to ω3-C18-PUFAs in galactolipids, SQDG and PG.

#### Impact of Overexpressing the Plastidial pω3-Des in the Native Host

##### FAs and Glycerolipids

*Ostreococcus tauri* lines overexpressing *p*ω*3-Des* (*p*ω*3-Des* OE) were created using the pOtOXLuc vector where the high affinity phosphate transporter promoter (PromHAPT) is driving transgene expression. The full activity of promHAPT requires phosphate limited conditions (Djouani-Tahri el et al., 2011). It should be recalled that phosphate deprivation triggers the increase of 18:2n6 and 18:3n3 at the expense 18:3n6 and 18:4n3 in plastidial lipids, most probably though the inhibition of the plastidial Δ6-desaturation (Degraeve-Guilbault et al., 2017, 2020).

**FIGURE 6:**
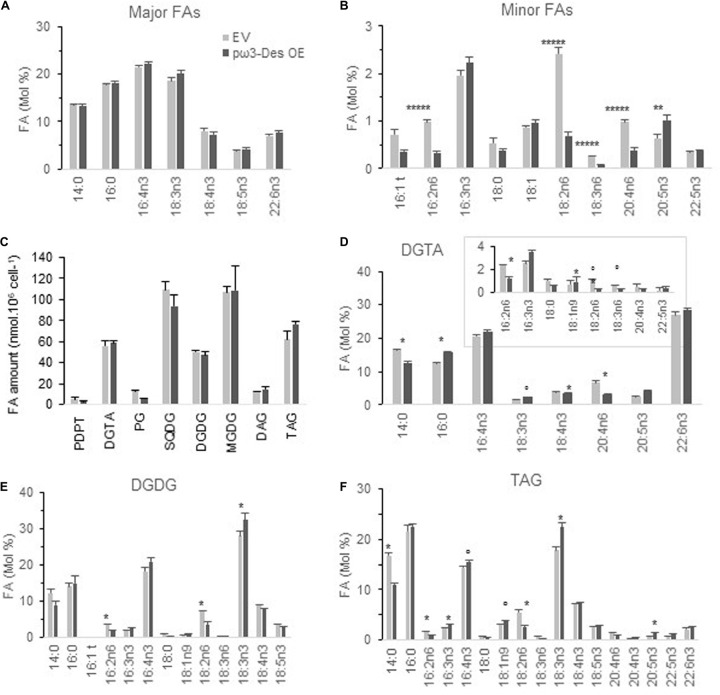
Glycerolipid profiles of *O. tauri* pω3-Des OE. **(A,B)** FA profiles of transgenics at stationary phase, 20°C. Means and standard errors are shown (*n* = 12 from three independent experiments and five pω3-Des OE different lines). Asterisks indicate statistically significant differences by Wilcoxon–Mann–Whitney test (**P < 0.005; *****P < 0.000001). Expression and luminescence levels of transgenics are available from Supplementary Figure 10. **(C)** Glycerolipid content of one pω3-Des OE line in mid-exponential growth. **(D–E)** FA profile of glycerolipids, DGTA **(D)**, DGDG **(E)**, and TAG **(F)**. Marks indicate statistically significant differences by *t*-test (°*P* < 0.02 and *P < 0.01). MGDG, SQDG are shown in Supplementary Figure 11. Means and standard errors from three independent experiments are shown.

From the five selected *p*ω*3-Des* OE subtle changes were detected under phosphate limitation at stationary phase (Figures 6A,B and Supplementary Figure 10). Proportions of all ω6-PUFAs were lower displaying relevant differences compared to control lines. As regards to minor ω3, 16:3n3 and 18:3n3 were slightly increased though only 20:5n3 appeared significantly higher. Variations in major ω3-PUFAs could, however, be detected from glycerolipids analysis (see below).

One of the transgenics was chosen for further detailed lipid analysis in mid-exponential growth (Figures 6C–F and Supplementary Figure 11). Glycerolipid composition of the *p*ω*3-Des* OE was not significantly altered. Conspicuous differences were observed in individual glycerolipids. A lower proportion of 14:0 and a higher proportion of 18:3n3 were more obvious in TAG, but also significant in DGTA (Figures 6D–F). The ω6 C16- and C18-PUFAs, especially 18:2n6, were reduced in plastidic and extraplastidic lipids including TAG, while both 16:3n3 and 16:4n3 were increased, though with poor significance. In DGTA 20:4n6 was reduced while all VLC-PUFAs downstream in the pathway were all slightly increased; this trend was also detectable in TAG. Altogether these changes are coherent with an increased ω3-desaturation activity in *p*ω*3-Des* OE that obviously impact all glycerolipids.

In summary, the overexpression the plastidial ω*3-Des* in the native host impacted the ω3/ω6 ratio at the level of minor FAs. These changes appeared to result from changes in all glycerolipid classes.

##### Growth at Different Temperature

FA remodeling of lipids is thought to be crucial for membrane acclimation to temperature changes. We therefore expected the growth of *p*ω*3-Des* OE to display some distinctive patterns when transferred to either lower or higher temperature (Supplementary Figure 12). The growth of *p*ω*3-Des* OE was, however, not consistently impacted neither by chilling nor by warming at limit high temperature (30–32°C).

## Discussion

In the present study, we achieved the functional characterization of the unique ω3-Des candidate from Mamiellophyceae species. Sequence analyses highlighted a predicted cTP, included in an ORF upstream of the conserved desaturase CDS, as well as His-Box motifs conserved in putative or established ω3-Des of protists belonging to the Chromista kingdom and highly distinctive from ω3-Des from cyanobacteria, fungi, plant, and animals. The Otpω3-Des localized to plastids in *N. benthamiana* and was shown to perform ω3-desaturation of all *Synechocystis* glycerolipid classes, which correspond to plastid lipids in eukaryotes. Moreover, *Otp*ω*3-Des* overexpression in the native host, though it poorly impacted the C18-PUFA pool, unambiguously affected the ratio ω6/ω3 of C16-PUFAs in DGDG, DGTA, and TAG and of VLC-PUFAs in extraplastidic lipids. These results are strongly recalling of features reported for the plastidial CrFAD7 which is the only ω3-Des in *Chlamydomonas.* Knocking-out or overexpressing *CrFAD7* impacted both plastidic and extraplastidic lipids suggesting that CrFAD7, located at the plastid envelope, may have access to extraplastidic substrates. This hypothesis applies for *O. tauri*. It is also possible that alternative translation occurs from the second methionine in the native host to produce an extraplastidic isoform (Kochetov, 2008). On the other hand, the overall weak phenotype of pω3-Des OE might be related to post-transcriptional control, at least in the native host, and/or to the importance of Δ6-Des activities for the fine-tuning of C18-PUFAs and thereof of downstream products. Post-transcriptional regulations of ω3-Des have been reported from cyanobacteria and plant and showed to even occur in heterologous systems (Sakamoto and Bryant, 1997; Matsuda et al., 2005; O’Quin et al., 2010).

### PUFAs and Temperature: Facts and Physiological Relevance

All organisms combined, the inverse correlation of FA unsaturation with temperature appears to be a universal trend. According to the homeoviscous hypothesis, the increase of FA unsaturation and thereby the decrease of the FA melting point, is necessary to maintain the fluid state of biological membranes at low temperature (Ernst et al., 2016). However, it should be emphasized that the addition of just one double-bound to saturated acyl-chain has the most drastic impact on the FA melting temperature and that the involvement of PUFAs for adjusting biomembrane physical properties has not been demonstrated; on the contrary omega-3 PUFAs failed to exhibit any peculiar fluidifying potency compared to oleic acid (De Santis et al., 2018). Although photosynthesis defects have been reported for mutants with altered UFAs content in plants and microalgae, it seems much more difficult to establish a causal link between these defects and impaired membrane fluidity (Vijayan and Browse, 2002; Falcone et al., 2004; Kugler et al., 2019). In cyanobacteria, ω3-PUFAs are only produced at chilling temperature and a direct link between membrane fluidity and *DesB* transcription has been demonstrated (Tasaka et al., 1996; Los et al., 2013; Mironov et al., 2018). However, neither membrane physical properties nor growth nor photosynthesis were significantly impacted in mutants lacking trienoic FAs. In contrast, plant mutants lacking trienoic FAs were reported to display distinct patterns of symptoms including sever thylakoid loss for cold temperature but also lower growth at high temperature (Routaboul et al., 2000). As trienoic PUFAs are the precursors of plant oxylipins and Nitro-FAs, it might be that these patterns are the result of impaired temperature signaling in plants (Mata-Perez et al., 2018; He and Ding, 2020; Yu et al., 2020). Though, establishing a relationship between PUFA membrane precursors and signaling down-products is a challenging issue, it should be kept in mind that oxylipins are also occurring in microalgae (Lauritano et al., 2016; Lupette et al., 2018).

As regards microalgae, temperature has been shown to impact FA profile in a species-dependent manner and it appears most difficult to discern a general trend from literature (Renaud et al., 1995; Boelen et al., 2013; Aussant et al., 2018; Gill et al., 2018; Balakrishnan and Shanmugam, 2020). Though it cannot be viewed as a general rule, the increase of ω3-PUFAs has been reported in response to chilling in various species of green microalgae while the increase of 18:5n3 and/or 20:5n3 was reported in Chromista species (see below) (Leblond et al., 2010; Kotajima et al., 2014; Aussant et al., 2018). The higher proportion of ω3 was in some case concomitant of a decline of the corresponding ω6-precursor (Nguyen et al., 2013; Zorin et al., 2017). The physiological relevance of PUFA variations have been only sporadically tackled. Works studying microalgal mutants with altered PUFA content at different temperature are scarce and overall provide mild evidences that mutations affect growth and/or photosynthetic processes (Sukenik et al., 1998; Nguyen et al., 2013; Zorin et al., 2017). For instance, the growth of *CrFAD7* knock-out mutant was not impaired at low temperature; extreme high temperature (45°C) was necessary to highlight that the mutation was associated with a reduced impairment of PSII activity. In these studies, early variations have not been investigated.

In the present work, experimental conditions were optimized for the identification of early temperature-specific FA variations. *O. tauri* growing temperature is commonly fixed at 20°C. In our conditions, *O. tauri* readily acclimated to 14°C and grew equally well at 24°C, in coherence with the range of temperature at which *Ostreococcus* species were identified in the environment (Limardo et al., 2017). Therefore, we can assume that temperature shifts between 14 and 24°C and reciprocally are moderate as regards to the thermoacclimation capacity of *O. tauri*. Finally, L/D entrainment was used to restrain internal time resetting by temperature reducing the chance that the differences observed are indirectly resulting from circadian rhythms shifts. It should be emphasized that early changes are more likely to be related to direct response to temperature whereas late variations may rather be related to indirect general metabolism adjustment. Chilling and warming triggered a swift and reverse adjustment of the ω6/ω3 PUFA ratio in all glycerolipids; the earliest variations occurred in C18-PUFAs (known to predominate in plastidic lipids), including 18:5n3, which is exclusively located in galactolipids (Degraeve-Guilbault et al., 2017). The progressive decrease of 20:4n6 upon chilling was also a robust trend. As evoked, compared to monounsaturated FA, the fluidizing potency of PUFAs have not been demonstrated to be better (De Santis et al., 2018). Though, the proportion of 18:1 is very low in *O. tauri* and even if it is increased in 14°C acclimated cells, other FAs, sterols and/or pigments might participate for acclimatizing membrane fluidity PUFAs are also known precursors of oxylipins and of Nitro-FAs in various organism ranging from microalgae to animals and their production could, to some extent, be related to the variation of membrane PUFAs and be involved in the signaling of temperature changes (Lupette et al., 2018; Wasternack and Feussner, 2018).

In the present work, the FA-phenotype of *p*ω*3-Des* OE was weak and it was therefore not surprising that no growth defect could be detected. We assume that either compensatory mechanisms are taking place in *p*ω*3-Des* OE and/or that our conditions are not appropriate and/or stringent enough to unveil any defects. Nevertheless, the fact that *p*ω*3-Des* OE displayed lipid features closely related to those of low temperature acclimated cells was obvious and coherent with the involvement of *p*ω*3-Des* in temperature acclimation. The reduction of 14:0 at low temperature might possibly be part of the homeoviscous process required at low temperature but in this case, would not be expected in the *p*ω*3-Des* OE.

### 18:5n3: An Enigmatic Marker of Temperature

Most interestingly, the 18:5n3 variations observed in *O. tauri* were reported in evolutionary distant microalgae (Renaud et al., 1995; Kotajima et al., 2014). As evoked, prasinophyta (including Mamiellophyceae) are the only primary endosymbiotic organisms that encompass 18:5n3, which is commonly found in microalgae emerging from secondary endosymbiosis. In the haptophytes, *E. huxleyi* and *Isochrysis* species 18:5n3 was reported to increase at low temperature while it was found at higher percentage (together with 18:4n-3) in cold-adapted dinoflagellates (Leblond et al., 2006). We show here that the content of 18:5n3 is tightly regulated by temperature in a species from the green lineage and varies as early as 6 h after temperature-shift. This result points out the involvement of this peculiar FA in temperature responses may be an ancient feature. Since 18:5n3 is predominantly located in glycerolipids across all species, this regulation might be related to the fine-tuning of photosynthesis. The increase of 20:5n3 upon chilling has been reported for several species for which 20:5n3 is a major component of galactolipids and further evidences suggested that it could be involved in photosynthesis, in particular in non-photochemical quenching in *Nannochloropsis gaditana CCMP526* (Renaud et al., 1995; Camacho-Rodriguez et al., 2013; Dolch et al., 2017; Gill et al., 2018). On the other hand, 20:5 derived isoprostanes were characterized from *Phaeodactylum tricornutum* (Lupette et al., 2018). Interestingly their content was inversely correlated to that of 20:5 FA. It is therefore possible that non-enzymatic oxylipin derived from pentaenoic FAs might be involved in a retrograde signaling for temperature stress.

Biosynthesis of 18:5n3 is an enigmatic issue; several hypotheses have been proposed including a yet not identified Δ3-Des or the shortening of 20:5n3 (Joseph, 1975). Though no Δ3-Des have been identified to date, a positional isomer of stearidonic acid encompassing a double bond at position three (18:4^Δ3,6,9,12^) in a thermophilic cyanobacterium has been described; as this species, like the bulk of cynaobacteria, do not produce VLC-PUFAs, this result is in favor of Δ3-Des activity in cyanobacteria (Rezanka et al., 2012). In *O. tauri*, the recent discovery of plastidial Δ6-desturases showed that the plastidic C18-PUFA-pool is regulated independently of ER-Des and therefore supports the existence of a yet not identified plastidial Δ3-Des in this species. This activity could be carried by either one of the plastidial Δ6-Des, as previously discussed elsewhere or by the Δ5-Des whose expression is tightly regulated by the temperature (Degraeve-Guilbault et al., 2020). It should be recalled here that *Bathycoccus prasinos* lacks both the *p*Δ*6-Des2* and 18:5 (Degraeve-Guilbault et al., 2017). On the other hand, several Δ5-Des from animals were reported to display extended Δ6 and Δ4 regiospecificities and plant Des regiospecificity was shown to switch with sub-cellular localization (Heilmann et al., 2004; Li et al., 2010). Noteworthy, a cTP is predicted for the Δ5-Des and the recent genomic sequence assembly from the *O. tauri* strain RCC1115 further pinpointed an additional ORF opening the possibility that alternative translation might be used to produce two differentially located isoforms of Δ5-Des (Hoffmann et al., 2008).

The 20:5 shortening hypothesis cannot be excluded and is supported for *O. tauri* by: (1) the existence of *sn-1/sn-2* 20:5/16:4 DGTA, (2) the occurrence of high level of 20:5 in the acyl-CoA pool and the detection of 18:5n3, and (3) the occurrence of 18:5 in DAGs (Degraeve-Guilbault et al., 2017). From these observations, it can be speculated that either 18:5-CoA is transferred to the plastid after 20:5-CoA shortening in the cytosol and specifically esterified to lyso-16:4 galactolipids, or that 18:5-DAG arising from remodeled DGTA 20:5/16:4 DGTA serve as precursors for 18:5-galactolipid synthesis. Until a Δ3-activity can be demonstrated the two hypotheses remain equally speculative.

### Temperature Control of Desaturase Expression

*Ostreococcus tauri* biological processes have been previously shown to highly rely on orchestrated transcriptional regulation (Monnier et al., 2010). Clustering of genes according to their temporal waveforms suggested that master transcriptional regulations are at work to coordinate lipid metabolism with chloroplast and carotenoids biogenesis at late night and with photosynthesis, oxidative stress, and DNA repair at mid-day. Transcriptional rewiring in response to temperature, especially chilling, has been extensively studied in freshwater cyanobacteria and plants and are beginning to be studied in marine cyanobacteria (Sinetova and Los, 2016; Shi et al., 2018; Breton et al., 2020; Guyet et al., 2020). However, there is a large gap of knowledge with regards to microalgae. Desaturase transcriptional induction in response to temperature and/or membrane fluidity has been demonstrated in freshwater cyanobacteria (Los et al., 1997). In *Arabidopsis*, the up-regulation of plastidial ω3-Des FAD8 upon cold/chilling was shown to involve both transcriptional and post-transcriptional regulations (Gibson et al., 1994; Matsuda et al., 2005). Higher transcript level of ω*3-Des* occurred in *Chlorella vulgaris* and *C. reinhardtii* at chilling temperature and early transcriptional activation of Δ*6-Des* has been reported for *Isochrysis sp*. (Suga et al., 2002; Nguyen et al., 2013; Wang et al., 2016). Part of our aim was to identify whether desaturases expression was regulated by temperature and how far these regulations were coherent with FA variations. Chilling was shown to swiftly and sustainably up-regulated most desaturase gene under both L/D and continuous light conditions while warming had an overall reverse impact. Under L/D, the plastidial desaturase genes *p*Δ*6-Des2* and *p*ω*3-Des* as well as Δ*5-Des* were the most up-regulated genes. Conversely, warming repressed *p*ω*3-Des, p*Δ*6-Des1*, *and*Δ*5-Des* and to a greater extent *Acyl-CoA-*Δ*6-Des.* These regulations are overall coherent with the activation of the ω3 pathway upon chilling and its repression upon warming. Since we previously showed that overexpression *Acyl-CoA*Δ*6-Des* in *O. tauri* mostly impacted TAG-FA profile, the temperature dependent regulation of *Acyl-CoA-*Δ*6-Des* expression might possibly be related to the accumulation of highly unsaturated TAG at low temperature. The differential transcriptional regulation of the plastidial Δ6-desaturases *p*Δ*6-Des1* and *p*Δ*6-Des2*, demonstrated to have different specificity for ω6 and ω3 might be required for proper adjustment of the ω3/ω6 ratio in response to temperature (Degraeve-Guilbault et al., 2020).

## Conclusion

The peculiar pentaenoic FA 18:5n3 and 20:5n3, that are dominating plastid FAs in microalgae, may be early and conserved marker of temperature acclimation. The involvement of ω3-Des in temperature acclimation, though clearly demonstrated in cyanobacteria and plants have been poorly illustrated in microalgae. Our result support that in *O. tauri*, the plastidial ω3-Des is involved in temperature acclimation and that Δ6-Des are further involved in fine-tuning C18-PUFAs. Only a few studies provided clues about the implication of these changes in cell physiology, mostly indicating a relationship between photosynthesis and PUFA. The selective pressure that ensured the maintenance of peculiar PUFAs in microalgae, most likely relies on multiple environmental cues making difficult to unveil defects in PUFA mutants using conditions limited to one or two parameters. An alternative, is to directly search for the molecular function of PUFAs in structuring and/or signaling.

## Data Availability Statement

The original contributions presented in the study are included in the article/Supplementary Material, further inquiries can be directed to the corresponding author/s.

## Author Contributions

CD-G performed most of the experimental work related to chilling and the associated (cloning, transgenic screening, HP-TCL, GC-FID, and RT-qPCR). NP created the *Synechocystis* DesB OE and Otpω3-Des OE (transformation and screening). MG performed the experimental work mostly related to warming including FAMES and transcriptional analyses as well as analyses of lipids from *Synechocystis* (HP-TLC, GC-FID, and RT-qPCR). CL performed the lipid analysis of the *O. tauri* Otpω3-Des overexpressor. FD performed the work and analyses on *N. benthamiana* (cloning, agro-transformation, and FAMES analysis). JJ performed the work on DES localization and RT-qPCR for chilling experiments. TK initially assessed the ω3-Des activity of *O. tauri* ortholog. IS supervised the work on *Synechocystis*. FC designed, supervised, and performed the research, analyzed the data (*O. tauri, N. benthamiana*, and *Synechocystis*), and wrote the manuscript. All authors contributed to the article and approved the submitted version.

## Conflict of Interest

The authors declare that the research was conducted in the absence of any commercial or financial relationships that could be construed as a potential conflict of interest.

## Footnotes

1 www.graphpad.com
