# Supplementary material for: Temperature Acclimation of the Picoalga Ostreococcus tauri Triggers Early Fatty-Acid Variations and Involves a Plastidial ω3-Desaturase
Source: Front Plant Sci. 2021 Mar 19;12:639330. doi: 10.3389/fpls.2021.639330 (PMC8018280; doi:10.3389/fpls.2021.639330)
Supplement: Supplementary file 1 [file Presentation_1.pdf]

A

Test	Temperature°C		Generation number
	initial	final	
10 vs 19°C*	19	10	0.05 °
	19	19	0.6
12°C vs 32°C	20	12	3.1
	20	32	2.05 °
12°C vs 22°C	20	12	0.76
	20	22	3.08
15 vs 25°C	20	15	2.84
	20	25	2.1

B

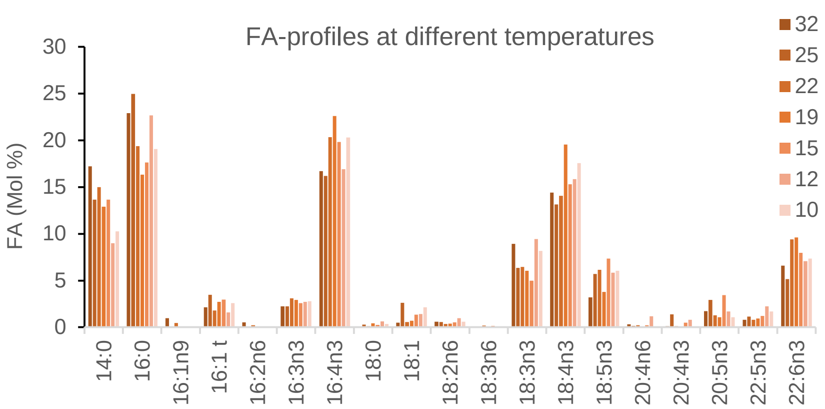

C D

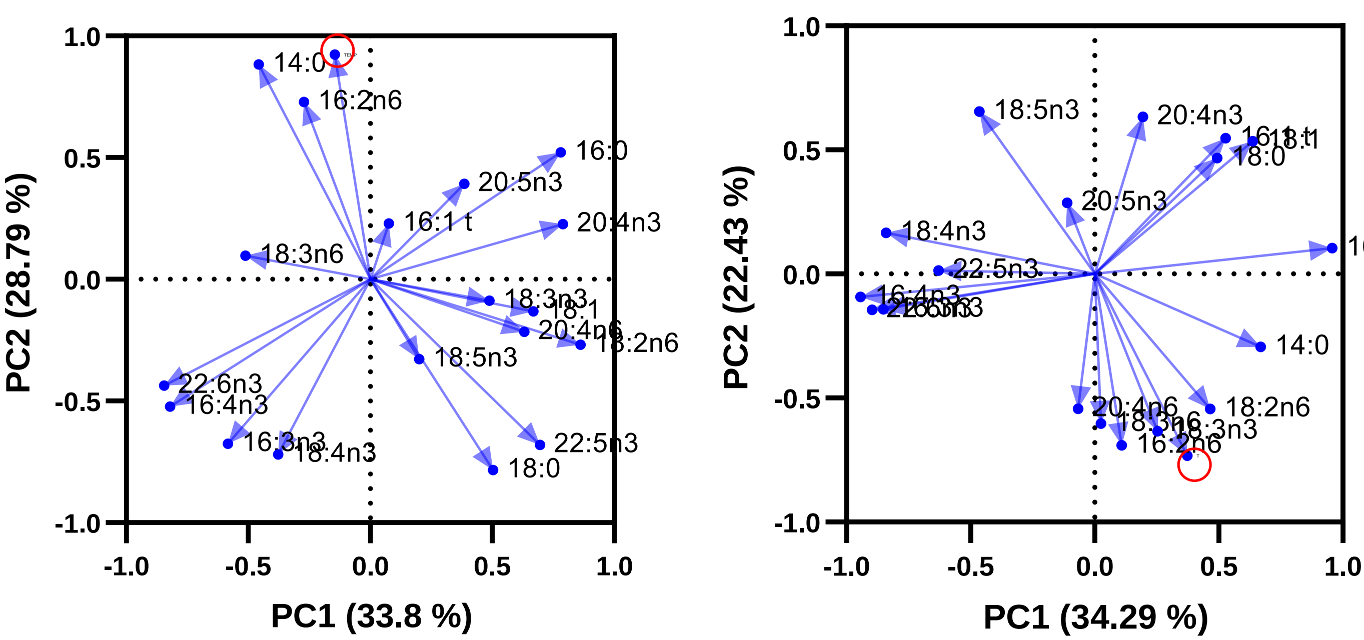

Supplemental figure 1. Temperature influence on FA profile.
Cells were acclimated to the initial temperature and sub-cultivated at different temperatures (test). **A.** Experimental conditions. Cells were acclimated at the initial temperature and sub-cultured (7.10^{6} cell.mL^{-1}) or transferred (7.10^{7} cell.mL^{-1}), label *, to the final temperature. Lethality after 24 to 48h at the final temperature is indicated by the symbol °. **B.** FA profile at the end of the experiment. **C-D**. Principal component analysis of FA and temperatures considering values at the end of the experiment (C) and all values collected during the kinetics after temperature shift (D). The variable temperature is highlighted by a red circle. Note that most ω6 FA variables displays correlation scores that are close to temperature in D while 18:5n3 shows an inverse correlation for both data sets.

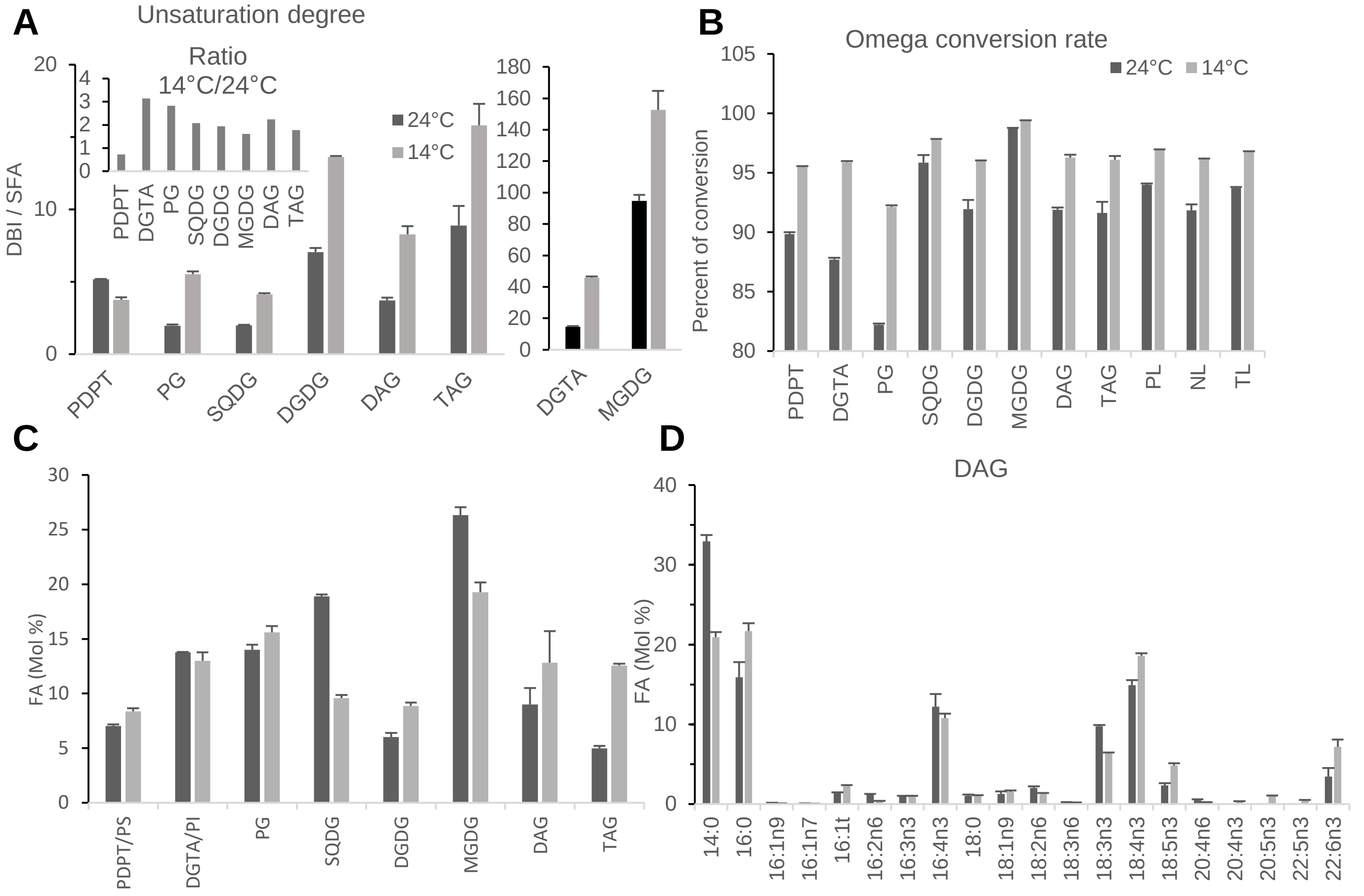

Supplemental figure 2. Glycerolipid FA profiles of cells acclimated at 24°C and 14°C. Complement to Figure 1. Note that PDPT, DGTA and PG contain a small proportion of PS, PI and PE respectively. **A.** Unsaturation degree of glycerolipids. The unsaturation degree is the ratio between the double bond index (DBI), that corresponds to the cellular molar proportion of double bonds, and the proportion of saturated fatty-acid (SFA). Variations at 14°C relative to 24°C are plotted in the upper left corner. **B.** Omega-3 conversion rate in glycerolipids. The values corresponds to the sum of ω3 divided by the sum of ω3 and ω6. In A and B, errors bars to the mean of ratio are shown (n=3). **C.** Glycerolipid profile in percent of total FA. **D.** DAG FA-profile.

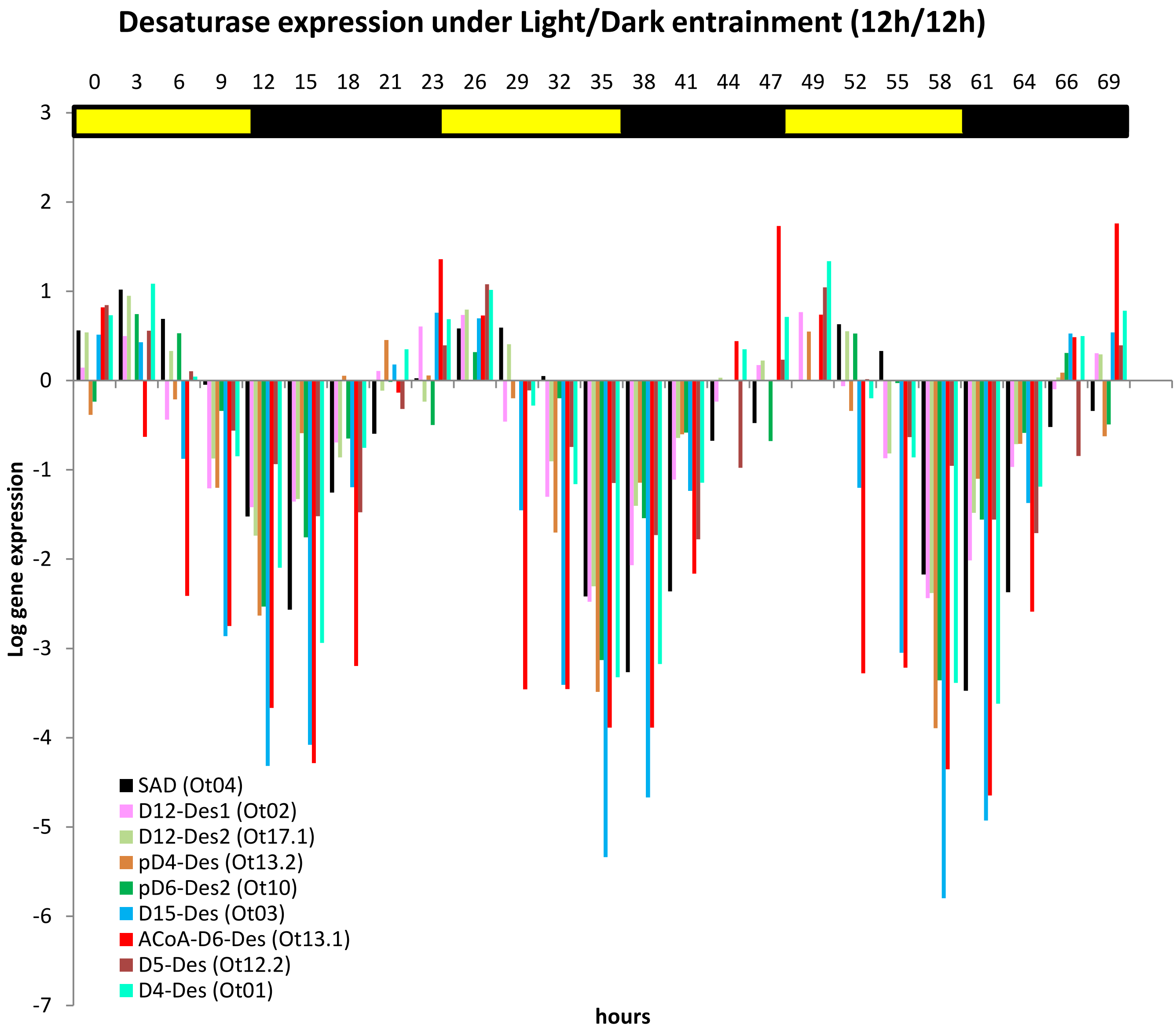

Supplemental figure 3. Raw data expression of *O. tauri* desaturases under diurnal conditions of 12h-light/12h-dark. Labels correspond to the putative or assessed desaturase activity and corresponding labels according to Degraeve et al. 2020 are in brackets. Original data were acquired by Monnier et al. (2010) and are available from the NCBI web site http://www.ncbi.nlm.nih.gov/geo/query/acc.cgi? token=jbmhpwkyccmuwvu&acc=GSE16422. Waveform expression clusters determined for some desaturases were the following: C4 (D12-Des, D4-Des, pD6-Des2), C7 (D5-Des), C1 (AcoAD7-Des). Note that for this previous experiment cultures were grown in vertical 250 mL flasks (T175 Sarstedt). The initial cell density was 2.5 10^6 cell.mL^{-1} and the doubling time was 24 h.

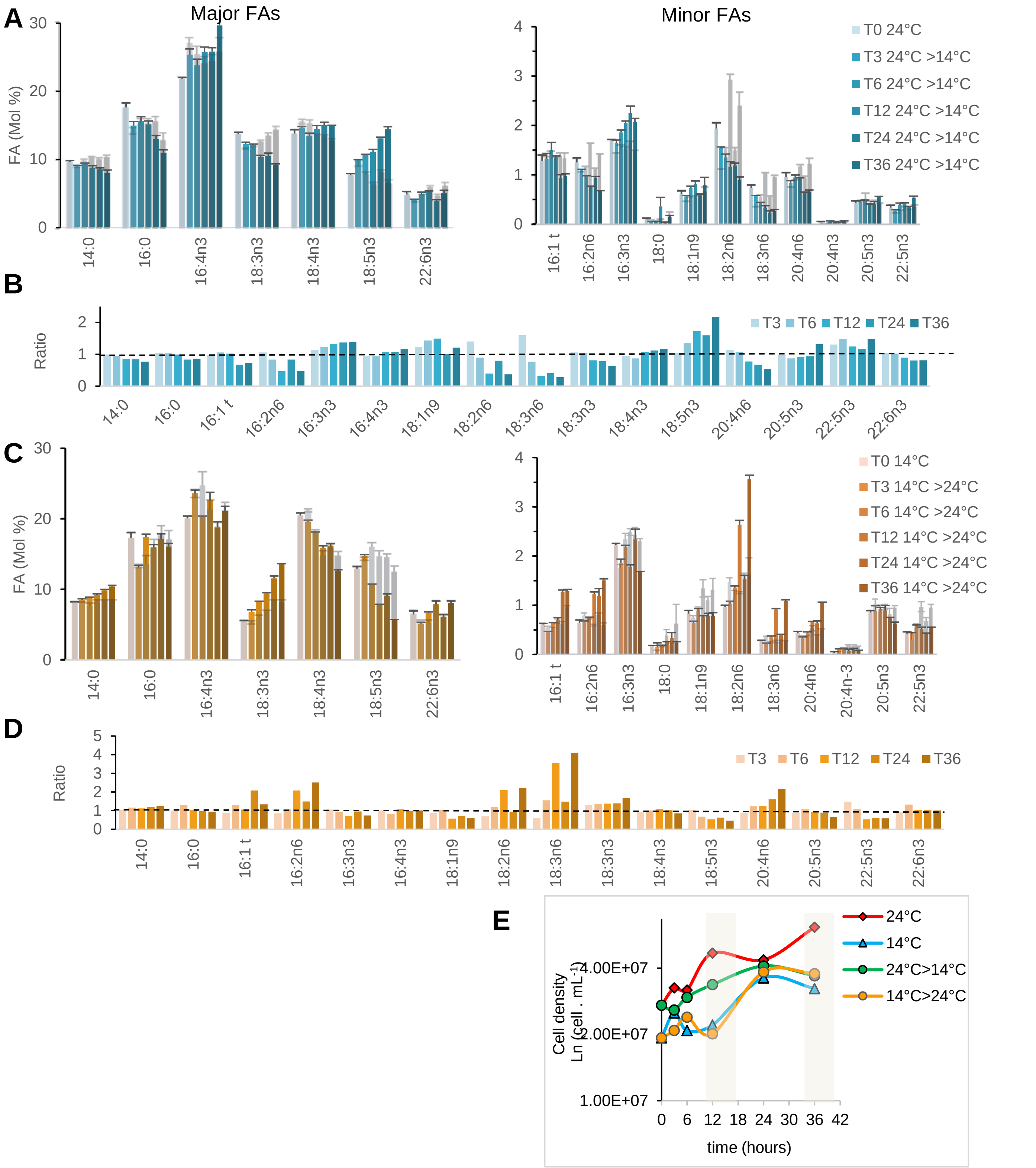

Supplemental figure 4. Kinetics of FA profile variations upon chilling and warming in one individual experiment.

Major and minor FAs are represented on separate panels. Cultures grown under light/dark cycles (16h/8h) at 24°C and 14°C were transferred at 4.5h after light on (T0) to 14°C (chilling, **A**) and 24°C (warming, **B**) respectively. The evolution of FA in control cells maintained at the initial temperature is shown in grey and ratio to the control at the initial temperature is further shown (**B** and **D**). Means and standard errors of independent biological triplicate are shown. **E.** Estimation of growth during the experiment.

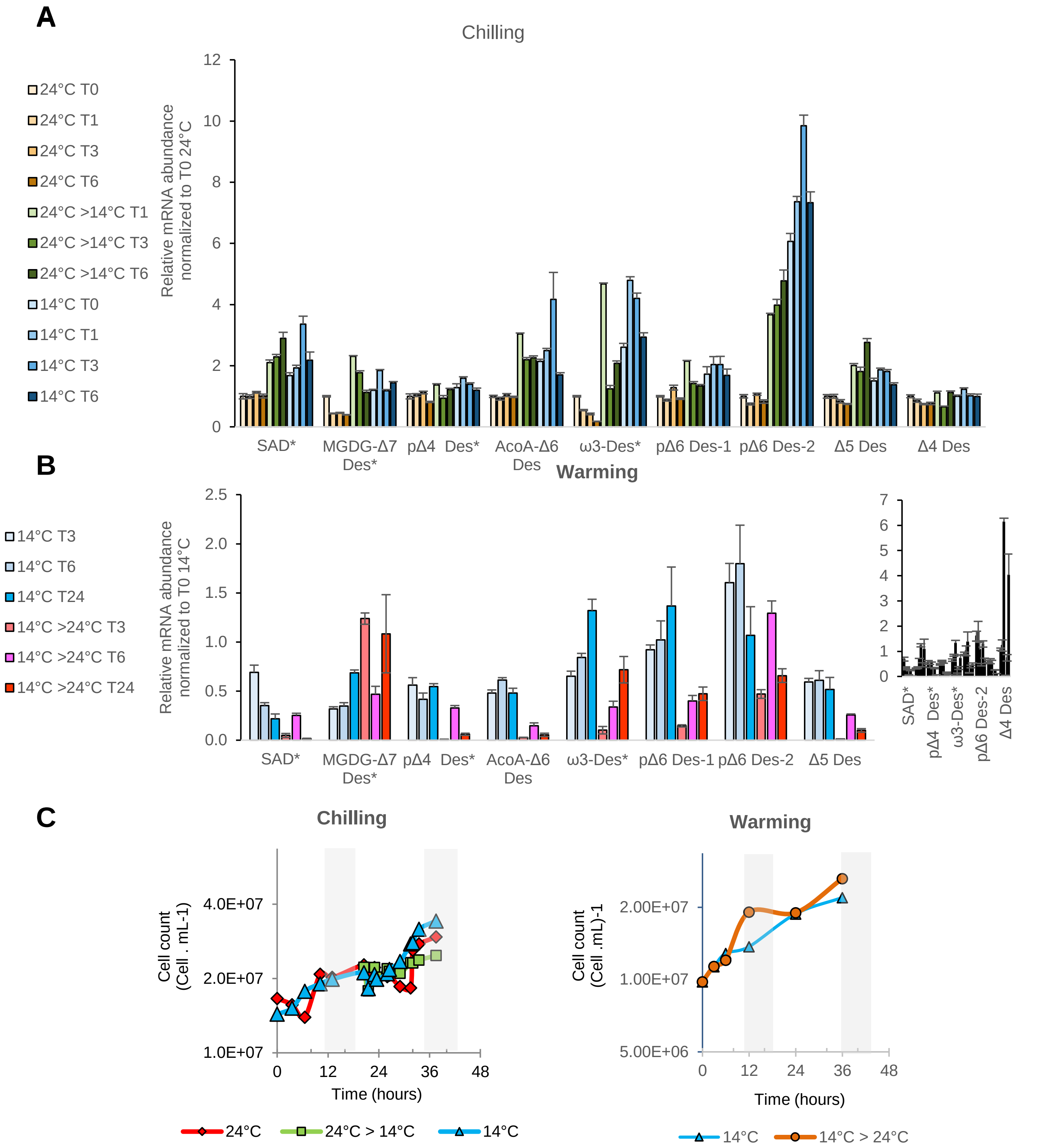

Supplemental figure 5. Transcriptional regulation of desaturases in different temperature conditions. Cells were grown under light/dark cycle 16h/8h and either maintained at the initial temperature or shifted to lower/higher temperature 4.5h after light on. **A**. Chilling shift. Expression in 24°C acclimated cells (orange bars) 14°C acclimated cells (blue bars) and chilled cells (green bars). T0 14°C was used for normalization (value=1,not shown). **B**. Warming shift. Expression in 14°C acclimated cells (blue bars) and cells transferred to 24°C (reddish bars). T0 24°C was used for normalization (value=1, not shown). Means and standard deviations of technical triplicate. **C**. Growing curves of corresponding cultures.

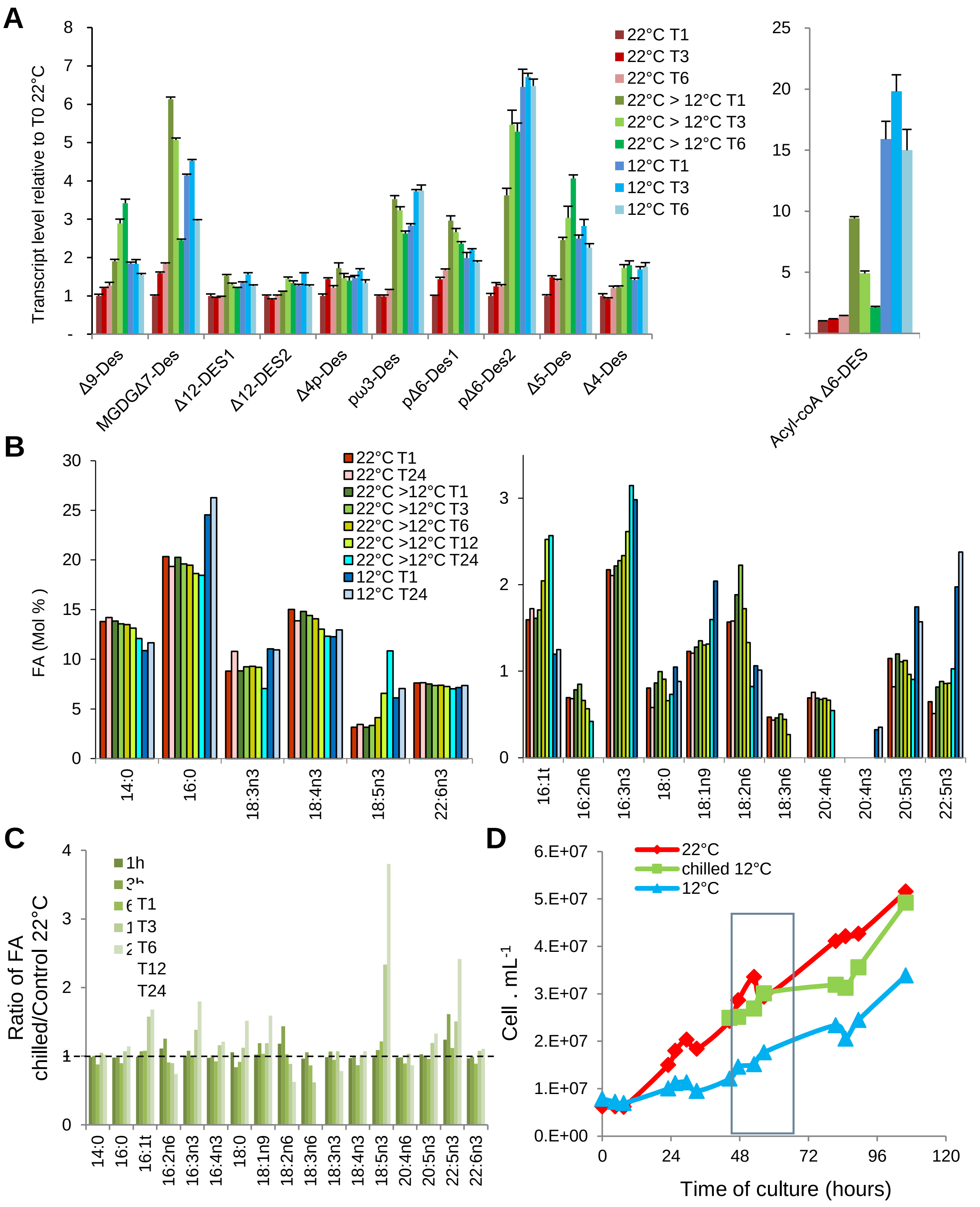

Supplemental figure 6. Desaturase transcript level upon chilling under continuous light. Cells were grown under continuous light and transferred from 22°C to 12°C (0h). Control cells were maintained at 22°C and 12°C. Control cells acclimated at 22°C (red bars) and 12°C (blue bars) and chilled cells (green bars). Means and standard deviations of technical triplicate are shown. **A**. Kinetics of desaturase expression changes. Means and standard deviations from technical replicate are shown. **B**. Corresponding FA-profiles. **C**. Corresponding variations **D.** Corresponding growth curves. Means of duplicate are shown. The period of sampling for RNA and FA analysis is highlighted by a frame.

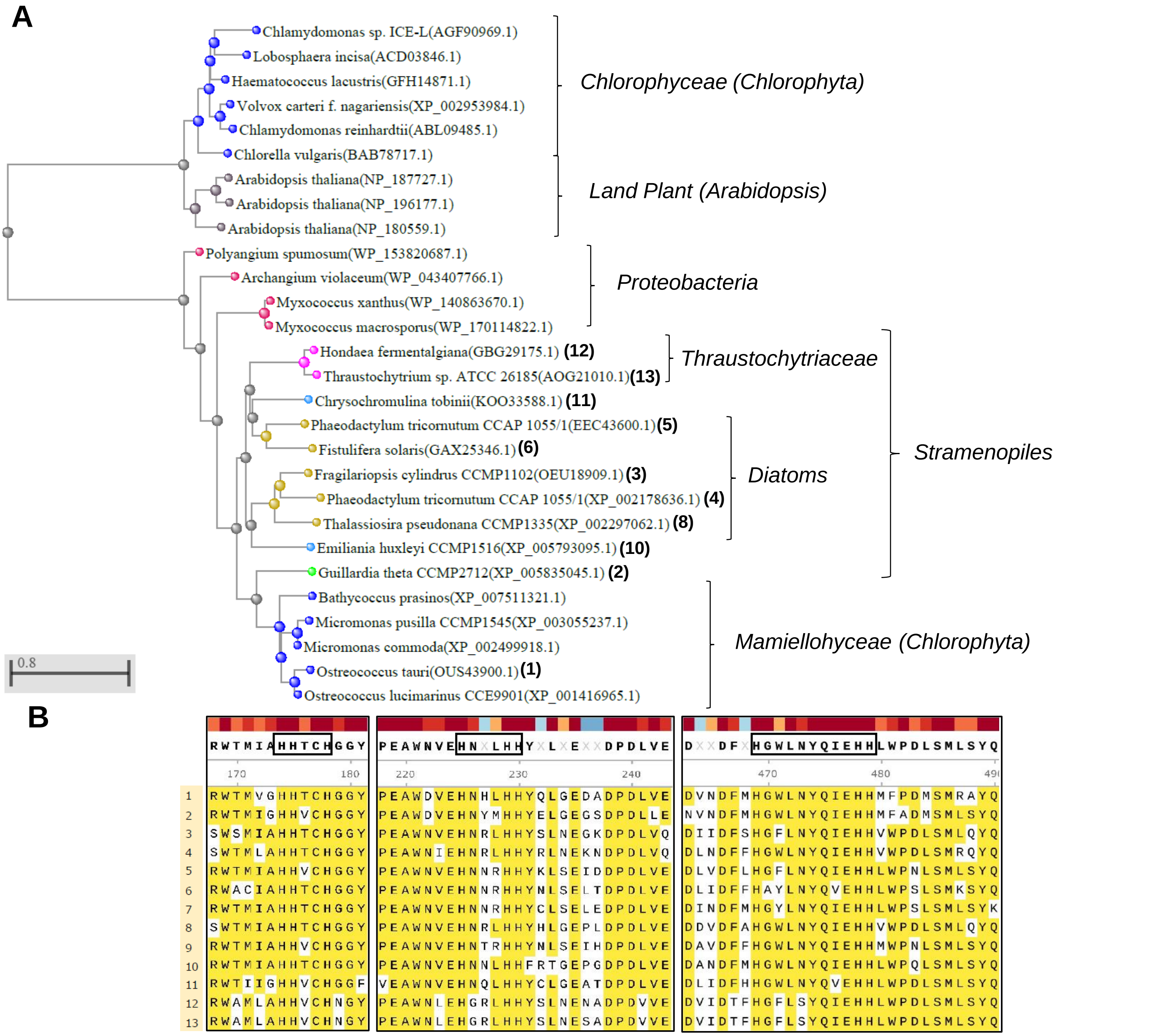

Supplemental figure 7. Sequences features of Mamiellophyceae ω3-Des candidates.

A. Phylogenetic tree of ω3-DES from microalgae and prokaryotes. (NCBI, COBALT). Tree method: fast minimum evolution. Distance: Grishin, Maximum sequence difference was increased to 0.9. to show ω3-Des from Chlorophyceae microalgae and plants species. Sequences of protein for which the activity was demonstrated were favored. Colors of nodes : eudicots (deep gray), green algae (deep blue), d-proteobacteria (red), haptophytes (light blue), cryptomonads (light green), slime nets (bright pink), diatoms (gold). Number in brackets are those of the sequences used the alignment. **B**. Histidine box regions Mamiellophyceae ω3-Des candidates. Color highlighting is based on conservation; consensus sequence and His-Box (frames) are shown. 1, ω3-Des (same as accession OUS43900.1); 2, XP_005835045.1 [Guillardia theta CCMP2712]; 3, OEU18909.1 hypothetical protein (hyp. Prot.) [Fragilariopsis cylindrus CCMP1102]; 4, XP_002178636.1 predicted protein (pred. prot.). [Phaeodactylum tricornutum CCAP 1055/1]; 5, EC43600.1 acyl desaturase [Phaeodactylum tricornutum CCAP 1055/1]; 6, GAX25346.1 hyp. Prot., [Fistulifera solaris]; 7, XP_002291233.1 pred. prot. [Thalassiosira pseudonana CCMP1335]; 8, XP_002297062.1 pred. prot. [Thalassiosira pseudonana CCMP1335]; 9, VEU34778.1 unnamed protein product [Pseudo-nitzschia multistriata]; 10, XP_005793095.1 Δ15 desaturase. EMIHUDRAFT_454147 [Emiliania huxleyi CCMP1516] (Kotajima et al, 2014); 11, KOO33588.1 hyp. prot. Ctob_010220 [Chrysochromulina tobinii]; 12, GBG29175.1 Fatty acid desaturase 3 [Hondaea fermentalgiana]; 13, AOG21010.1 omega3 desaturase [Thraustochytrium sp. ATCC 26185].

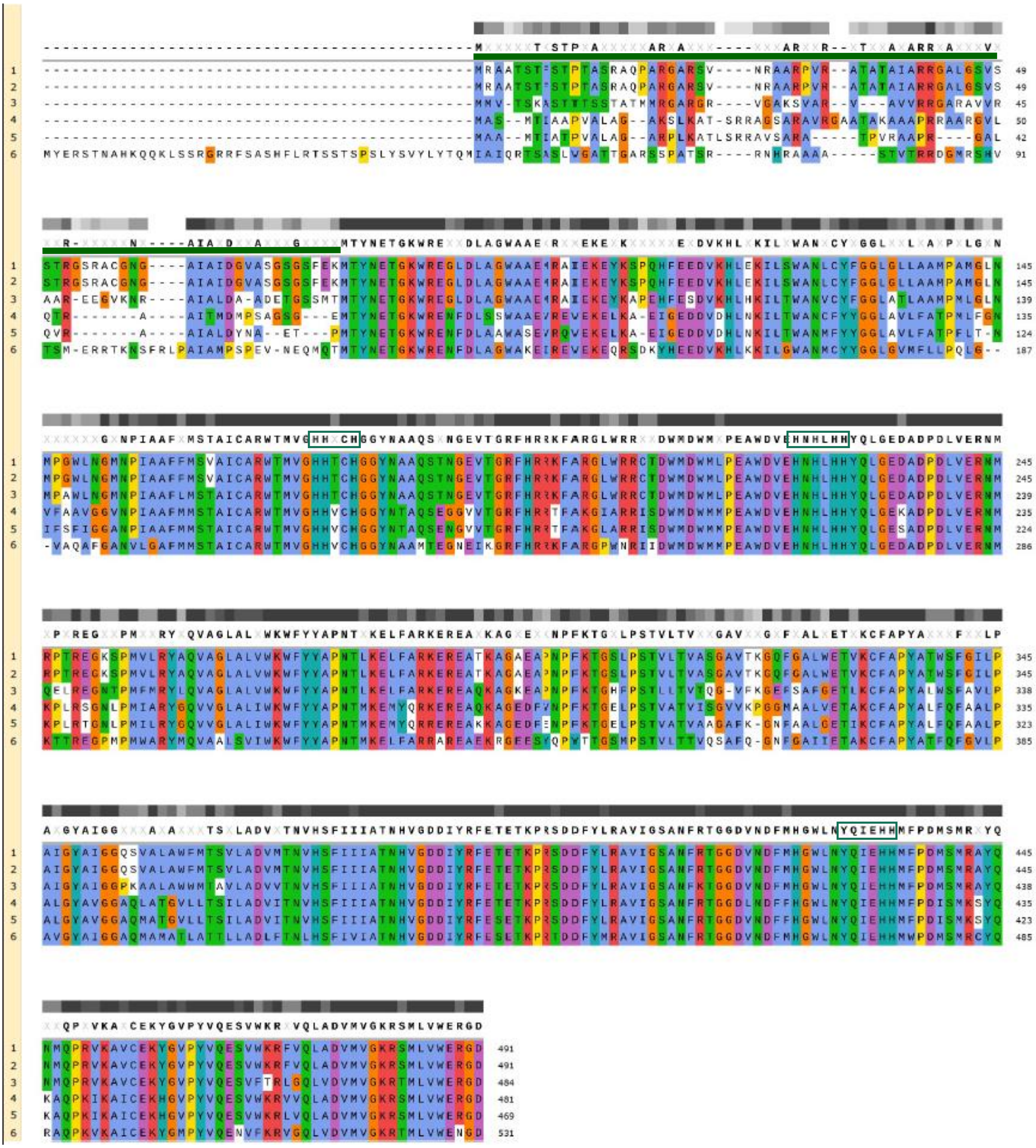

Supplemental figure 8. Sequence alignment of ω3-Des candidates from Mamiellophyceae. 1. Ostreococcus tauri (XP_003078215.2) extended in Nt and validated by mRNA amplification in this work. 2. Ostreococcus tauri (OUS43900.1). 3. Ostrococcus lucimarinus (XP_001416965.1) extended in Nt. 4. Micromonas pusilla (XP_003055237.1). 5. Micromonas commoda (XP_002499918.1) extended in Nt. 6. Bathycoccus prasinos (XP_007511321.1). The Nt extensions corresponding to putative cTP according to PredAlgo is highlighted by a green line.

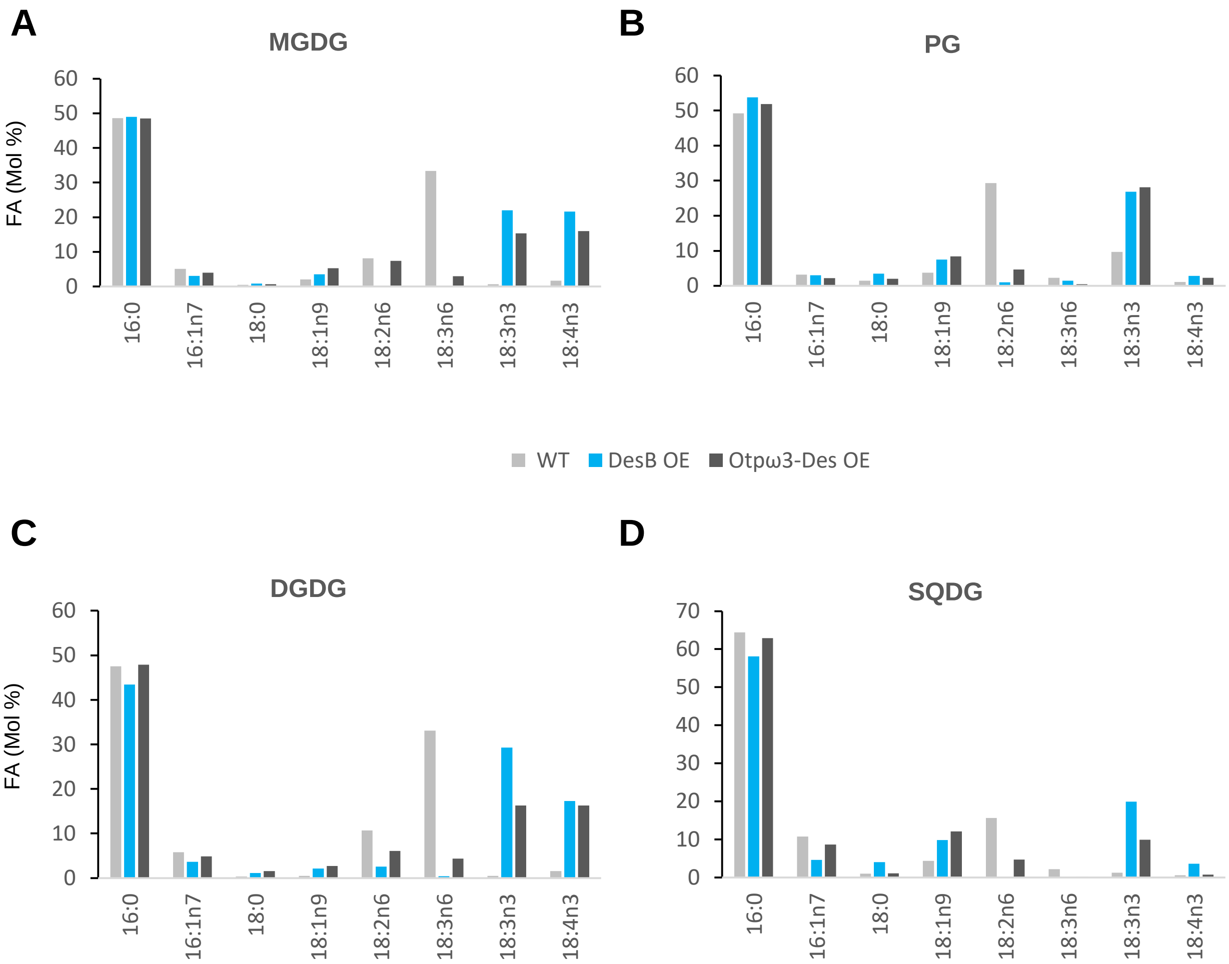

Supplemental figure 9. Glycerolipid FA profile of *Synechocystis* PCC6803 overexpressing of the *O. tauri* ω3-Des (Otpω3-Des) and the native ω3-Des (DesB OE). Cells were grown at 32°C. Biological duplicate were pooled for the analysis.

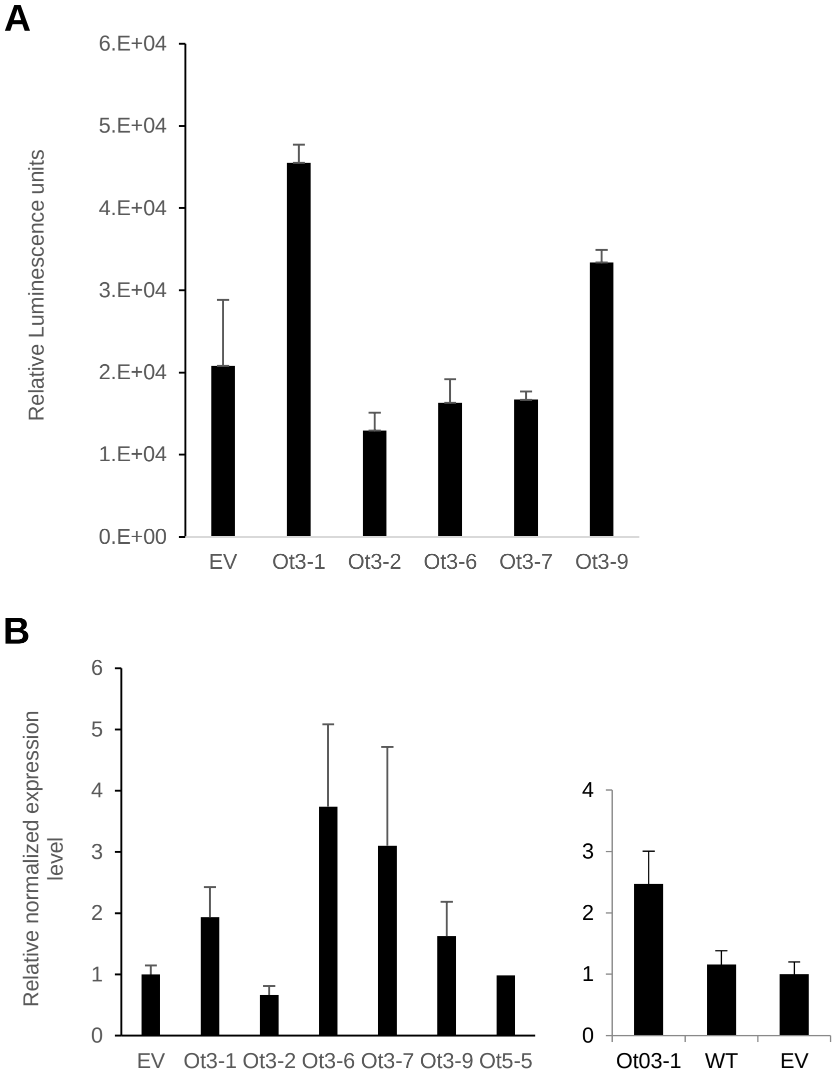

Supplemental figure 10. Omega-3-Des expression in *O. tauri* pω3-Des transgenics.
A. Luminescence of pω3-Des transgenics**.** The luciferase gene is expressed from the CCA1 promoter. Luminescence from 200µL of cells at stationary phase. **B**. ω3-Des transcript abundance in selected *pω3-Des* transgenics (Ot3-x), *empty-vector* transgenics (EV) and the *pΔ6-Des1* overexpressor as supplemental control (Ot5-5). An independent experiment is further shown for the Ot3-1 line on the right panel. This line was chosen for detailed glycerolipid analysis. Means and standard deviations of technical triplicate are shown.

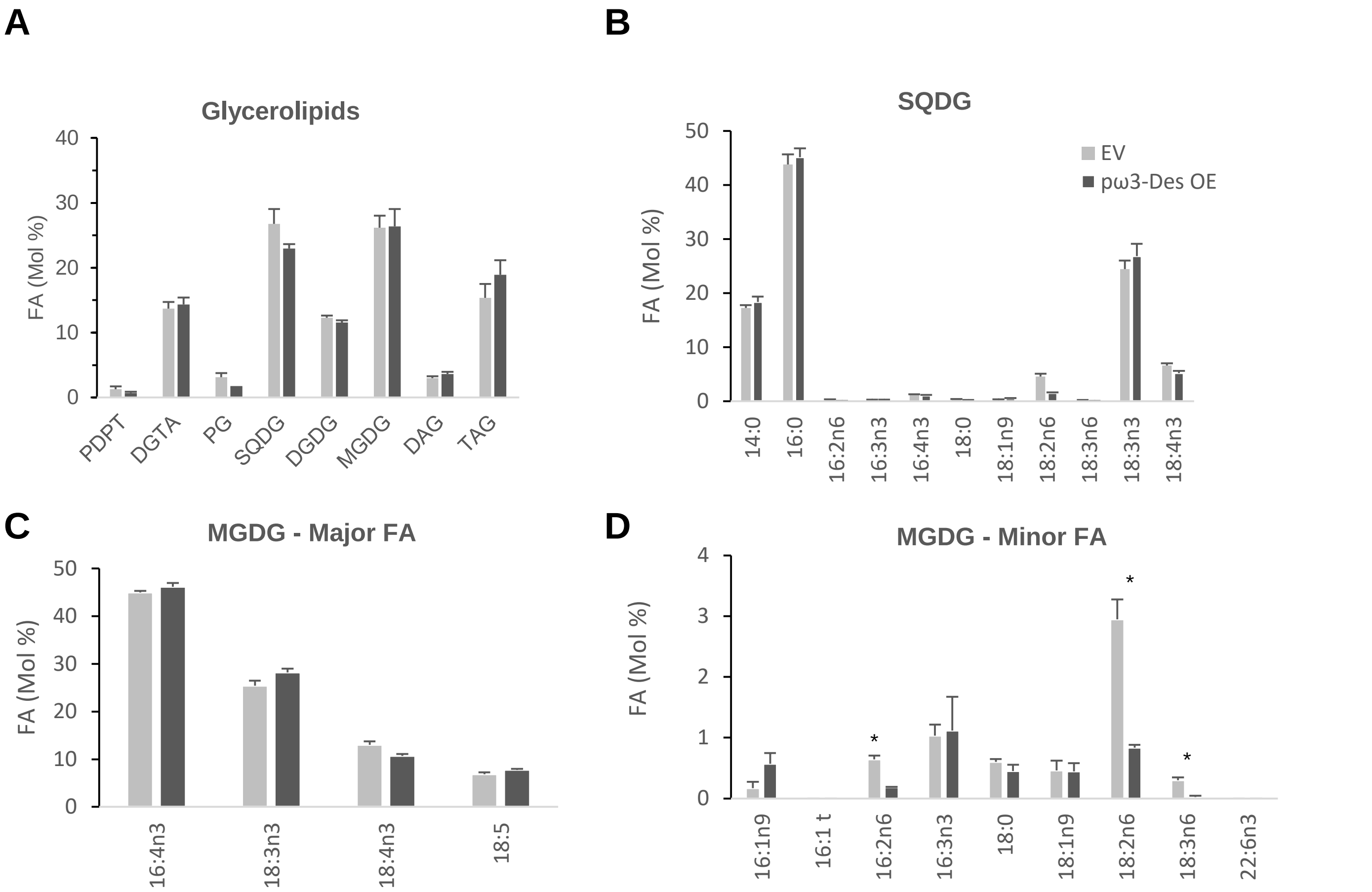

Supplemental figure 11. Glycerolipid FA profile of *O. tauri* overexpressing of the *O. tauri* ω3-Des (pω3-Des). Means and standard errors of 3 independent experiments are shown.

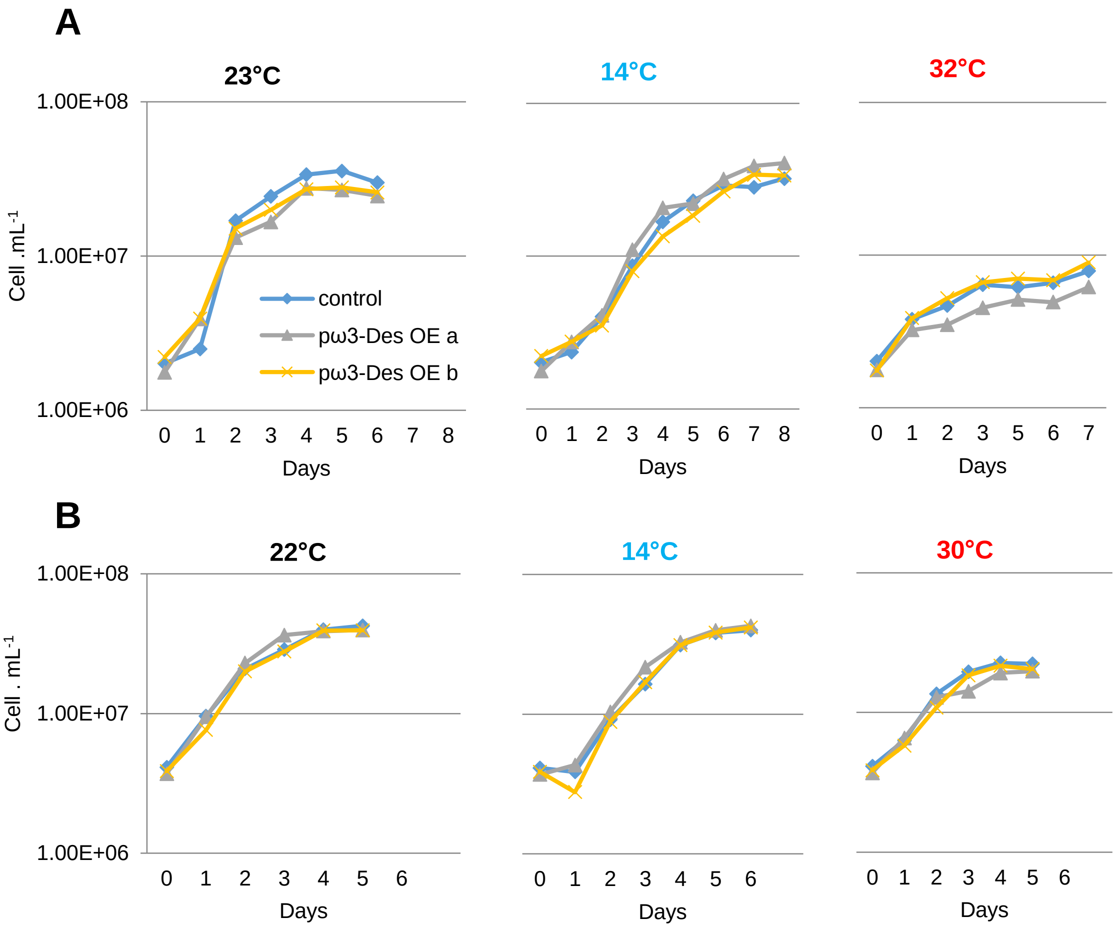

Supplmental figure 12: Growth curves of selected desaturase overexpressors shifted to low and high temperature.

Cells grown under continuous light, were acclimated at 22°C and 23°C. A) cells were subculture at the initial temperature and at 14°C and 32°C. C) Cells were sub-cultured at the initial temperature and at 14°C and 30°C. The experiment was stopped after stationary phase establishment. Means of duplicate are shown in each experiment.

Primer name	SEQUENCE 5'-3'	Cloning	Comments
Ot03g03040ApaI-fw	GGGCCCATGCGCGCCGCGACGTC	pOtoxLuc	amplified from pGEMT-easy suncloning
Ot03g03040AvRII-rv OK	CCTAGGCTAGTCGCCCCGCTCCCAGAC	pOtoxLuc	amplified from pGEMT-easy suncloning
attB1Ot03g03040-FR	GGGGACAAGTTTGTACAAAAAAGCAGGCTAAATGCGCGCCGCGACG	Gateway	
attB2Ot03g03040-FR	GGGGACCACTTTGTACAAGAAAGCTGGGTGGTCGCCCCGCTCCCAG	Gateway	ORF localisation no stop
Ot03g03040-FOR	ATGCGCGCCGCGACGTCG	pGEMT-easy	Used for cDNA amplification
Ot03g03040-REV	CTAGTCGCCCCGCTCCCAG	pGEMT-easy	

Supplemental Table 1. Primers for cloning the *O. tauri* ω3-desaturase ORF in the different vectors.

GENE locus tag	target	Primer name	SEQUENCE 5'-3'	Porduct length
Ot14g03380	Actine like prot	OtActProt2qPCR-fw	ATCGTTCGGCGGATATGGAG	98
		OtActProt2qPCR-rev	CGTCTCTCGCGCCAATTGTA	
Ot18g00850	Calmodulin	Cal Fw	CGGGATGTTTTTGCGGTGTT	96
		Cal Rev	CGTCAATTTCTCCCCCACGA	
Ot04g05560	EF1	EF1-Fw	GTGCCGTGATAAAGCCGAAC	158
		EF1-Rev	GCCGACTGCCATCGTTTTAC	
Ot04g03720	SAD	P4551	TTCGCTTTCTTCCCGACCTC	80
		P4552	CTGAACATTCCGCAGTTGGC	
Ot17g02260	MGDGΔ7-Des	P4555	GAGTAAGGCTGAGGGTCCCG	81
		P4556	CACCTGTCGTGTTGCCAGA	
Ot13g01600	pΔ4-Des	P4565	TTTAGCGTTCCCGTTGTTGC	84
		P4566	AGCTTAGCGCCTTCGGTATC	
Ot05g00190	pΔ6-Des1	P4543	CCCTTCGCGGAAAAGAATGG	93
		P4544	GCTTGAGCGTTCGAAACACC	
Ot10g02610	pΔ6-Des2	P5903	CGATGGTTCTGGGGTGGAAT	165
		P5904	GATGCCTTCGAGATGACGGT	
Ot03g03040	pω3-Des	P4549	CCGCGTGTCAAGGCTGTCTG	98
		P4550	TTGCCAACCATGACGTCCGC	
Ot13g01070	AcoA-Δ6-Des	P4537	TCGACATCGATCCGAGTCAAG	80
		P4538	CGGAAAGAGGTGGTGGATGA	
Ot01g00780	Δ4-Des	P4539	AATCGAGCACCACCTCTTCC	82
		P4540	TTTCGCACACTCGTCCTTGA	
Ot12g02780	Δ5-Des	P4557	GTCGTCCACCATCTCTTCCC	80
		P4558	TTCTCAGCTGCCGCCTTAAT	

Supplemental Table 2. Primers for qPCR.
